# Dopant-Free Ultra-Thin Spiro-OMeTAD Enables Near 30%-Efficient n–i–p Perovskite/Silicon Tandem Solar Cells

**DOI:** 10.1007/s40820-025-01962-3

**Published:** 2026-01-14

**Authors:** Xiangying Xue, Weichuang Yang, Zhiqin Ying, Fangfang Cao, Yuheng Zeng, Zhenhai Yang, Xi Yang, Jichun Ye

**Affiliations:** 1 Zhejiang Provincial Engineering Research Center of Energy Optoelectronic Materials and Devices, Ningbo Institute of Materials Technology and Engineering, Chinese Academy of Sciences (CAS), Ningbo, 315201 People’s Republic of China; 2 Research Center for Wide Bandgap Semiconductors and Devices, YongJiang Laboratory, Ningbo, 315202 People’s Republic of China; 3 School of Optoelectronic Science and Engineering & Collaborative Innovation Center of Suzhou Nano Science and Technology, Key Lab of Advanced Optical Manufacturing Technologies of Jiangsu Province & Key Lab of Modern Optical Technologies of Education Ministry of China, Soochow University, Suzhou, 215006 People’s Republic of China

**Keywords:** Perovskite/silicon tandem solar cells, Hole transport layers, Optical loss reduction, Optical design

## Abstract

A major challenge for n–i–p structured perovskite/silicon tandem solar cells (TSCs) is the use of 2,2′,7,7′-tetrakis[*N,N*-di(4-methoxyphenyl)amino]-9,9′-spirobifluorene (spiro-OMeTAD), a commonly used hole transport layer, which induces significant optical losses and consequently reduces device current. Herein, we propose an ultra-thin (10 nm) vacuum thermal evaporation (VTE)-deposited spiro-OMeTAD, coupled with a 2D/3D perovskite heterojunction, to simultaneously enhance the optical and electrical properties of n–i–p perovskite/silicon TSCs. Our results demonstrate that the 10-nm-thick spiro-OMeTAD layer significantly improves optical performance, achieving a 92.2% reduction in parasitic absorption and an 18.4% decrease in reflection losses. Additionally, the incorporation of the 2D/3D perovskite heterojunction facilitates improved molecular arrangement and enhanced surface uniformity of the ultrathin spiro-OMeTAD, leading to higher tolerance to interface defects and more efficient hole extraction. Consequently, n–i–p perovskite/silicon TSCs featuring ultrathin spiro-OMeTAD exhibit remarkable efficiencies of 29.73% (0.135 cm^2^) and 28.77% (28.25% certified efficiency, 1.012 cm^2^), along with improved stability.

**Supplementary Information:**

The online version contains supplementary material available at 10.1007/s40820-025-01962-3.

## Highlights

- Optical loss reduction: The 10-nm-thick vacuum thermal evaporation (VTE)-based 2,2′,7,7′-tetrakis[*N,N*-di(4-methoxyphenyl)amino]-9,9′-spirobifluorene (spiro-OMeTAD) film reduces parasitic absorption by 92.2% and reflection losses by 18.4% compared to conventional spin-coated 200-nm-thick spiro-OMeTAD.
- Electrical performance improvement: The VTE-deposited spiro-OMeTAD, coupled with a 2D/3D perovskite heterojunction, ensures conformal coverage, optimizes energy-level alignment, and passivates interface defects.
- Device efficiency and stability enhancement of perovskite/silicon tandems: A remarkable efficiency of 29.73% is achieved, the highest reported value for spiro-OMeTAD-based n–i–p tandems, with a 11 times improvement of stability under operational conditions.

**Supplementary Information:**

The online version contains supplementary material available at 10.1007/s40820-025-01962-3.

## Graphical abstract

**Figure:**
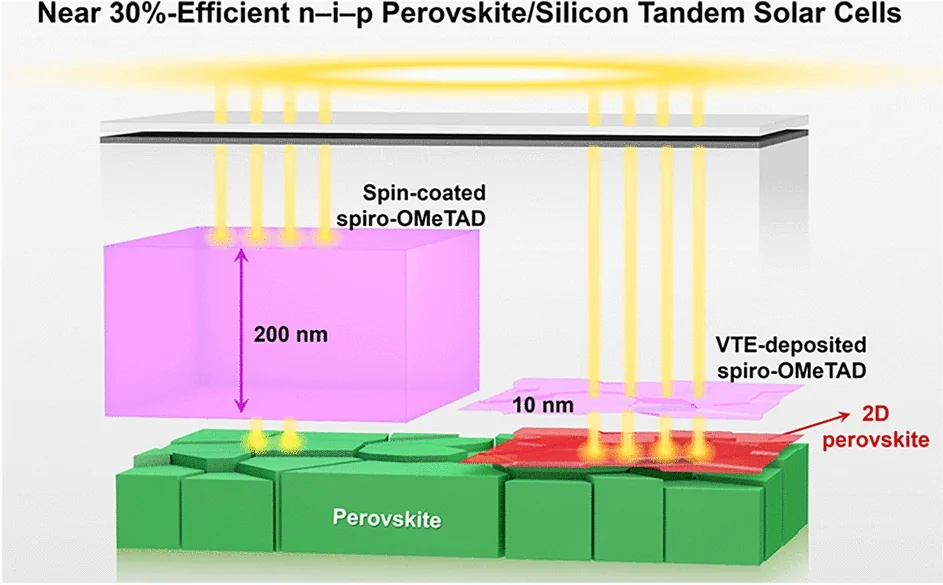

## Introduction

Perovskite/crystalline silicon (c-Si) tandem solar cells (TSCs) are emerging as a promising photovoltaic (PV) technology, having recently achieved a record power conversion efficiency (PCE) of 34.85% [1]. However, the achievement of high PCEs (i.e., > 30%) is predominantly realized in inverted (p–i–n) configurations [2–9], whereas the best-performing regular (n–i–p) TSCs exhibit a PCE of only 29.2% [10]. Theoretical predictions suggest a maximum PCE of 42.5% for perovskite/c-Si TSCs, irrespective of the device configuration [11, 12]. The performance gap between these two configurations primarily results from differences in short-circuit current density (*J*_SC_), with p–i–n perovskite/c-Si TSCs achieving a *J*_SC_ exceeding 21 mA cm^−2^, whereas their n–i–p counterparts reach only 19.8 mA cm^−2^ [10, 13]. This discrepancy highlights the critical need for enhanced optical optimization in n–i–p perovskite/c-Si TSCs, which is currently hindered by optical parasitic losses in the commonly used hole transport layer (HTL) material, 2,2′,7,7′-tetrakis[*N,N*-di(4-methoxyphenyl)amino]-9,9′-spirobifluorene (spiro-OMeTAD), despite its success in single-junction perovskite devices with outstanding PCEs [14–16]. Additionally, to ensure uniform film coverage and minimize leakage current, spiro-OMeTAD is usually deposited using the spin-coating method with a thickness of 100–200 nm [17, 18], resulting in significant parasitic absorption across the entire spectral range, particularly at the wavelengths of 300–500 nm. Moreover, spiro-OMeTAD induces strong reflection, especially in the infrared region (800–1000 nm), further limiting the current in the silicon bottom sub-cells [19]. Therefore, optimizing the optical properties of the HTL is crucial for achieving efficient n–i–p perovskite/c-Si TSCs.

To address this challenge, several strategies have been explored. One approach involves modifying or replacing spiro-OMeTAD with alternative HTL materials such as poly(triaryl amine) (PTAA) to form a composite HTL, which can mitigate the negative optical impact of spiro-OMeTAD while maintaining efficient hole transport [20, 21]. Another strategy focuses on reducing the HTL thickness. Recently, Ye et al. developed a minimalist transparent hole-selective contact by incorporating cross-linkable *p*-type small molecules into the antisolvent, which significantly enhanced *J*_SC_ of n–i–p perovskite/silicon TSCs, achieving a certified efficiency of 29.2% [10]. Furthermore, wide-bandgap inorganic materials, such as NiO_*x*_, offer potential benefits, including improved optical performance and reduced sputtering damage during transparent electrode deposition [22]. However, interfacial recombination losses caused by energy level misalignment between perovskite and NiO_*x*_ can negatively impact open-circuit voltage (*V*_OC_). Notably, vacuum thermal evaporation (VTE) has emerged as a promising strategy for precisely controlling the thickness of spiro-OMeTAD [23–26]. This fine-tuned control of HTL thickness enables simultaneous reduction in parasitic absorption and enhancement of light management while maintaining compatibility with various substrates for improved electrical properties [23, 24]. However, challenges such as incomplete coverage of VTE molecules (leading to leakage currents) and the need for dopants often necessitate thicker HTL layers [25], limiting their optical performance. Moreover, the co-evaporation process requires complex fabrication equipment. Therefore, further efforts are urgently needed to optimize the trade-off between optical and electrical performance in HTLs for n–i–p perovskite/c-Si TSCs, which represents a key factor in unlocking their full PCE potential.

This work presents an application of an ultrathin (10 nm) dopant-free VTE-deposited spiro-OMeTAD HTL facilitated by a 2D/3D perovskite heterojunction as a strategy to address optical losses in conventional thick spiro-OMeTAD layers, thereby enhancing the *J*_SC_ and PCE of n–i–p perovskite/c-Si tandem solar cells. Post-treatment of the perovskite film with an* n*-butylammonium iodide (BAI) solution induces a capping layer of BA-based 2D perovskite on the 3D perovskite, thus forming a 2D/3D perovskite heterojunction. This approach serves a dual purpose: firstly, it facilitates the conformal coverage of the ultrathin spiro-OMeTAD layer over the perovskite film. Secondly, it promotes favorable energy level alignments and defect passivation, effectively suppressing nonradiative recombination at the spiro-OMeTAD/perovskite interface. Our findings demonstrate that a 10-nm-thick spiro-OMeTAD is sufficient for efficient hole extraction, reducing parasitic absorption by 92.2% and reflection losses by 18.4% compared to conventional 200-nm-thick films. Consequently, semitransparent perovskite solar cells with such a spiro-OMeTAD achieve an impressive efficiency of 17.40% when illuminated from the HTL side. Furthermore, the perovskite/c-Si TSCs featuring the thin spiro-OMeTAD exhibit a remarkable efficiency of 29.73% (certified at 28.25%), representing the highest reported value for n–i–p perovskite/c-Si TSCs with spiro-OMeTAD. Notably, the absence of dopants in the VTE-deposited spiro-OMeTAD significantly enhances the stability of TSCs, maintaining 88% (80%) of its initial PCE after 1000 h (110 h) of illumination under maximum power point tracking conditions. These findings show the potential of ultrathin HTLs for realizing highly efficient and stable perovskite/c-Si TSCs.

## Experimental Section

### Materials

Perovskite precursors were fabricated using methylammonium bromide (MABr) (GreatCell Solar Materials), formamidinium iodide (FAI) (GreatCell Solar Materials), cesium iodide (CsI) (Sigma Aldrich), lead bromide (PbBr_2_) (TCI), and lead iodide (PbI_2_) (TCI) powders. The electron transport layer (ETL) was fabricated using a SnO_2_ colloid precursor (tin oxide, 12% in H_2_O colloidal dispersion) (Xi’an Yuri Solar Co., Ltd.), while the HTL was fabricated with spiro‐OMeTAD (Xi’an Yuri Solar Co., Ltd.). Solvents such as *N,N*-dimethylformamide (DMF), dimethyl sulfoxide (DMSO), and chlorobenzene (CB) were sourced from Sigma-Aldrich. Pre-patterned ITO glass was obtained from Advanced Election Technology Co., Ltd., and the metal electrode deposition mask was custom-made by Shenzhen Rigorous Technology Co., Ltd.

### Fabrication of the Perovskite Solar Cells

The perovskite solar cells in the single junction and tandem configurations were fabricated on ITO and silicon heterojunction (SHJ) substrates, respectively. For ITO, the substrates were cleaned sequentially in a cleaning solution, deionized (DI) water, acetone, and ethanol, each under 15 min of ultrasonication, followed by drying in a vacuum oven. Subsequently, the cleaned substrates were treated with UV-ozone for 15 min. A film of tin oxide (SnO_2_) was deposited by spin coating at 3000 rpm for 30 s and annealed at 150 °C for 30 min. The SnO_2_ solution was prepared by diluting the tin oxide solution in DI water at a 1:3 ratio. Next, 40 µL of perovskite solution, with a composition according to reference literature, was spin-coated at 3000 rpm for 40 s. In the last 20 s of spinning, 250 µL of chlorobenzene (CB) was used for antisolvent extraction, followed by annealing at 100 °C for 20 min. The 2D perovskite layer was prepared by spin coating with a BAI solution (2 mg mL^−1^) at 5000 rpm and annealing at 100 °C for 10 min. The HTL layer of the target device was a 10-nm spiro-OMeTAD. The spiro-OMeTAD layers were deposited on the perovskite layer via VTE; the evaporated speed was 0.1 Å s^−1^ at the vacuum condition (Starts at *p* < 5 × 10^–4^ pa, works at *p* < 1 × 10^–3^ pa). Because of the slow evaporation rate of spiro-OMeTAD, at the very beginning we warmed the crucible at 10 °C intervals with a target temperature of 350 °C. Upon reaching 350 °C, temperature variations within ± 5 °C All depositions were monitored with quartz crystal microbalance crystals maintaining > 90% sensitivity to ensure thickness control. No additional oxidation process was applied to the VTE-deposited spiro-OMeTAD devices before the efficiency evaluation. In the control device, the spiro-OMeTAD HTL was spin-coated onto the perovskite layer at 3000 rpm for 30 s. The spiro-OMeTAD solution consists of 73.5 mg spiro‐OMeTAD, 25 µL tBP, 17 µL Li-TFSI (520 mg in 1 mL acetonitrile), and 8 µL FK 209 Co(III) TFSI (360 mg in 1 mL acetonitrile), stirred overnight. A 10-nm-thick molybdenum oxide layer was deposited by thermal evaporation as a buffer layer, followed by the deposition of a 50-nm-thick indium zinc oxide (IZO) through PVD. Finally, a 200-nm-thick gold electrode was deposited by thermal evaporation.

### Fabrication of Monolithic Perovskite/Silicon Tandem Solar Cells

The perovskite top cells were fabricated using a similar procedure to that of single-junction devices, but with a few modifications. Following the deposition of SnO_2_, an aqueous solution of KCl with a concentration of 0.8 mg mL^−1^ was spin-coated at a speed of 3000 rpm for 30 s, followed by annealing at 100 °C for 10 min in ambient air. In particular, we increased the concentration of the perovskite solution from 1.5 M to 1.7 M. The thickness of the gold electrode was increased to 300 nm. Finally, a 110-nm-thick magnesium fluoride (MgF_x_) layer was deposited by e-beam deposition as an anti-reflection coating.

### Measurements and Characterization

The field-emission scanning electron microscope (SEM) (Hitachi, S4800) was utilized to capture SEM images using an electron beam accelerated at 4 kV. The surface of the samples was coated with Pt to improve surface conductivity to prevent charge accumulation during the measurement. Ultraviolet photoelectron spectroscopy (UPS) and X-ray photoelectron spectroscopy (XPS) measurements were conducted using a Kratos AXIS ULTRA DALD, with UPS measured under the He I (21.22 eV) emission line at a 5 V bias voltage. An ultraviolet–visible spectrophotometer (PerkinElmer) recorded the absorbance spectra. X-ray diffraction (XRD) employed Cu Kα radiation on Bruker AXS D8 Advance across a range of 3°–35°. The Xeuss Waxs/Saxs System (Xenocs, France) (1.54 Å) collected Grazing Incidence Wide-Angle X-Ray Scattering (GIWAXS) patterns; the angle of incidence is 0.1°. Kelvin probe force microscopy (KPFM) images were observed on a scanning probe microscope (Dimension ICON, Bruker, USA). Time-Resolved Photoluminescence (TrPL) decays were induced by FluoTime 300 (PicoQuant) with a 450-nm laser; TrPL excitation was incident from the perovskite side. Transient absorption spectroscopy (TAS) was measured by SOL-F-K-HP-T at 800 nm. Steady-state photoluminescence (PL) spectra were observed by a fluorescence spectrometer (HORIBA, FL3-111) with an excitation wavelength of 470 nm. The current–voltage characteristics of the devices were measured by a Keysight B2901A Source Meter under AM 1.5G illumination with a solar simulator (Enlitech, SS-F5-3A) as the light source. The intensity of the light was calibrated with a KG-5 filter Si photodiode. And the single-junction perovskite solar cells were measured with a metal mask (0.135 cm^2^) to reduce the influence of the scattered light. The measuring condition for single-junction perovskite solar cells was forward scan and reverse scan (1.2–0.0 V, scan rate 100 mV s^−1^, delay time 10 ms). The measuring condition for perovskite/c-Si TSCs was forward scan and reverse scan (0.0–2.0 V, scan rate 100 mV s^−1^, delay time 10 ms). The EQE spectra were recorded in direct current (DC) mode using an Enli Technology (Taiwan) standard single-crystal Si and Ge reference solar cells were used to calibrate. During the measurement of the perovskite top sub-cell, the silicon bottom sub-cell was saturated with continuous bias light from a white light lamp equipped with a long pass (> 800 nm) filter. During the measurement of the silicon bottom sub-cell, the perovskite top sub-cell was saturated with continuous bias light from a white light lamp equipped with a low pass (< 540 nm) filter. The stability of perovskite/c-Si TSCs was monitored using the same equipment as the PCE test. The stability of single-junction perovskite solar cells was monitored under continuous light soaking (xenon lamp).

### Simulation Methods

In this study, the two-dimensional finite element method by coupling optical and electrical models was used [27]. The optical simulation was carried out by solving Maxwell’s equations to obtain the frequency-dependent and spatial-dependent electromagnetic distributions. In that case, the optical properties including optical absorption efficiency and optical generation rate can be thus obtained. The incident light with wavelengths ranging from 300 to 1200 nm was considered. The wavelength-dependent optical constants (i.e., the refractive index* n* and extinction coefficient *k*) were all extracted from references [28, 29]. To quantitatively evaluate the device effect, *J*_ph_ values of the absorption efficiency (Abs) in the perovskite layer of incident light weighted by AM1.5G were expressed as follows:

1 $$J_{{{\mathrm{ph}}}} = \int\limits_{{300{\mathrm{nm}}}}^{{800{\mathrm{nm}}}} {\frac{q\lambda }{{hc}}} {\mathrm{Abs}}(\lambda )\Phi_{{{\mathrm{AM}}1.5}} (\lambda )d\lambda$$

where *q* is the unit charge, *h* is the Plank’s constant, *c* is the speed of light in vacuum, and *Φ*_AM1.5_ is the solar spectral irradiance under AM1.5G. Similarly, current density of the reflection efficiency (*R*) and the parasitic absorption were assessed using the same method.

Based on the carrier generation in the optical simulation, the electrical simulation was then implemented to carrier dynamics processes including carrier recombination, transport and collection. By addressing the Poisson’s and drift–diffusion equations of the electrical simulation, the electrical parameters of PSCs can be obtained. Here, the simulated parameters used for this simulation, which included the optical input, i.e., refractive indexes of the related materials, and electrical input, i.e., electron affinity, bandgap, permittivity, effective conduction/valence band density, doping concentration, mobility as well as various types of recombination coefficients, etc., were extracted from the previous works [27]. In detail, a set of typical Poisson’s, carrier continuity, and drift diffusion equations were solved to obtain the performance of the PSCs, given by following equations:

2 $$\nabla \cdot \left( { - \varepsilon_{r} \nabla V} \right) = q\left( {p - n + N_{{\mathrm{d}}} - N_{{\mathrm{a}}} + n_{{\mathrm{t}}}^{ + } - n_{{\mathrm{t}}}^{ - } } \right)$$

3 $$\frac{\partial n}{{\partial t}} = \frac{1}{q}\nabla \cdot J_{{\mathrm{n}}} + G - R;\;\frac{\partial p}{{\partial t}} = - \frac{1}{q}\nabla \cdot J_{{\mathrm{p}}} + G - R$$

4 $$J_{{\mathrm{n}}} = qn\mu_{{\mathrm{n}}} \xi + qD_{{\mathrm{n}}} \nabla n;\;J_{{\mathrm{p}}} = qp\mu_{{\mathrm{p}}} \xi - qD_{{\mathrm{p}}} \nabla p$$

where *ɛ*_r_ is dielectric constant of semiconductor, *V* is the electrostatic potential, *n* (*p*) is the electron (hole) concentration, *N*_d_ (*N*_a_) is the ionized donor (acceptor) concentration, *n*_t_^+^(*n*_t_^−^) is the donor (acceptor) trap density, *J*_n_ (*J*_p_) is the electron (hole) current density, *μ*_n_ (*μ*_p_) is the electron (hole) mobility, *D*_n_ (*D*_p_) is the electron (hole) diffusion coefficient, which can be deduced by Einstein’s relation, *i.e.*, *D*_n,p_ = *μ*_n,p_*k*_B_*T*/*q* (*k*_B_ is the Boltzmann’s constant, *T* is the operating temperature, which was fixed at 300 K in this simulation), *ξ* is the electric field that is extracted by *ξ* =  − ∇*V*, *∂n*/*∂t* (*∂p*/*∂t*) is transient electron (hole) concentration gradient, which was fixed at 0 because only the steady-state case was considered here. *R* is the total carrier recombination rate, which can be classified into three types of recombination mechanisms:

5 $$R= {R}_{\mathrm{rad}}+{R}_{\mathrm{Aug}}+{R}_{\mathrm{SRH}}+{R}_{\mathrm{sur}}$$

6 $${R}_{\mathrm{rad}}={B}_{\mathrm{rad}}(np-{n}_{\mathrm{i}}^{2})$$

7 $${R}_{\mathrm{Aug}}=({A}_{\mathrm{n}}n+{A}_{\mathrm{p}}p)(np-{n}_{\mathrm{i}}^{2})$$

8 $${R}_{\mathrm{SRH}}= \frac{np-{n}_{i}^{2}}{{\tau }_{\mathrm{p}}\left(n+{n}_{\mathrm{t}}\right)+{\tau }_{\mathrm{n}}(p+{p}_{\mathrm{t}})}$$

9 $${R}_{\mathrm{sur}}= \frac{np-{n}_{i}^{2}}{\left(n+{n}_{\mathrm{ts}}\right)/{S}_{\mathrm{n}}+(p+{p}_{\mathrm{ts}})/{S}_{\mathrm{p}}}$$

where *R*_rad_ is the radiative or direct recombination rate, *R*_Aug_ is the Auger recombination rate, *R*_SRH_ is the Shockley–Read–Hall recombination rate, *R*_sur_ is the surface recombination rate, *B*_rad_ is the radiative recombination coefficient, *A*_n_ (*A*_p_) is the electron (hole) Auger recombination coefficient, *τ*_n_ (*τ*_p_) is the electron (hole) lifetime, *S*_*n*_ (*S*_p_) is the surface recombination velocity of electron (hole), *n*_i_ is the intrinsic carrier concentration, *n*_t_/*n*_ts_ (*p*_t_/*p*_ts_) is the bulk/surface electron (hole) concentration of the trap state. *R*_SRH_ and *R*_sur_ can be also expressed by interface and bulk defect densities (*D*_it_ and *N*_t_), respectively, *i.e.*, *D*_it_ = *v*_th_*σ*_n,p_*S*_n,p_, *N*_t_ = 1/(*v*_th_*τ*_n,p_*σ*_n,p_), where *v*_th_ is the thermal velocity and *σ* is the capture cross section of the states. A good estimation was presumed by approximating the bulk and interface states of all energies into a single energy at the middle level of bandgap, thus *n*_t_ = *n*_ts_ = *p*_t_ = *p*_ts_ = *n*_i_. The electrical periodic boundary conditions in the horizontal direction were applied.

## Results and Discussion

### Optical Regulation and Photovoltaic Performance

**Fig. 1:**
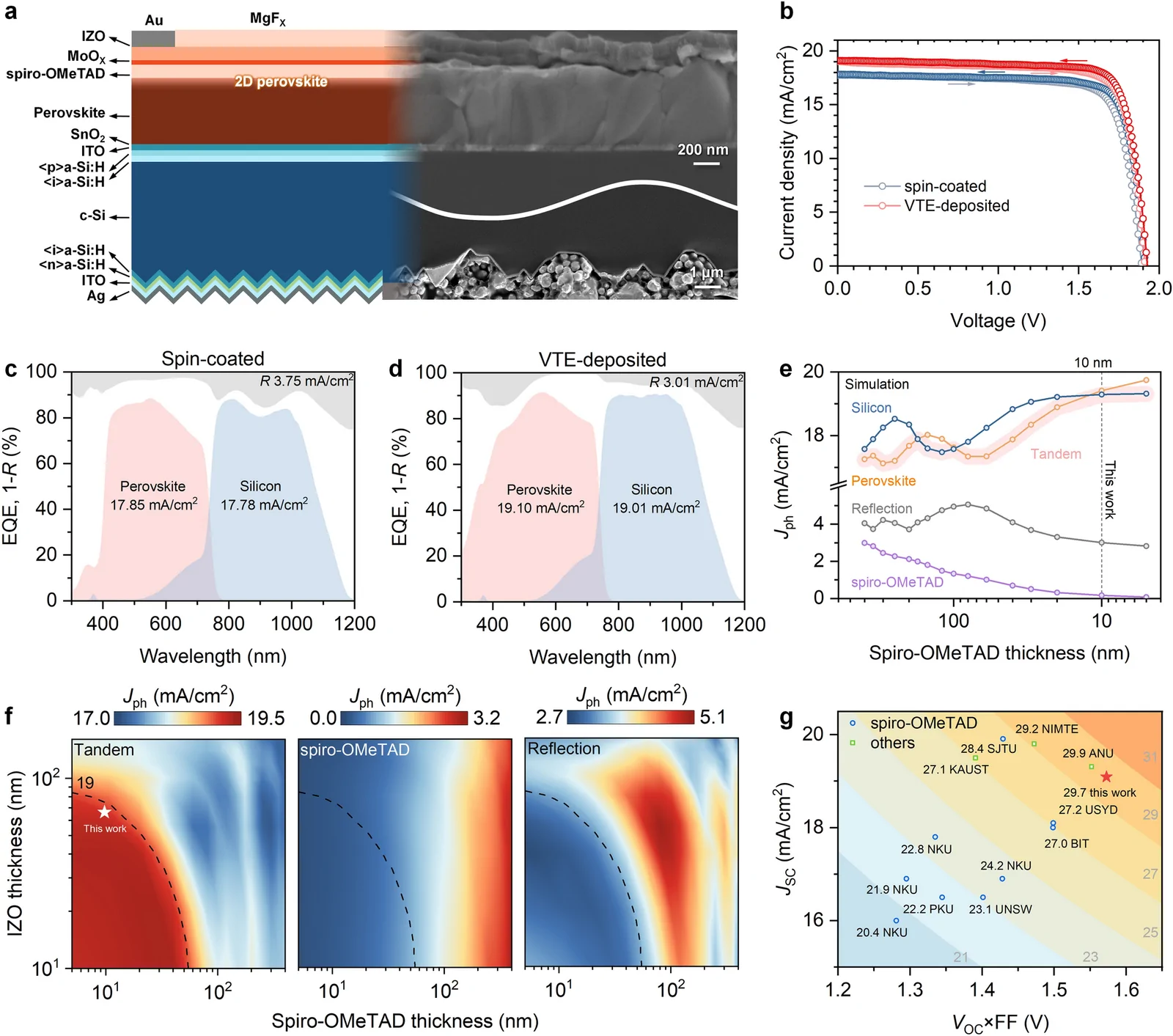
Photovoltaic performance and optical response of TSCs.** a** Schematic diagram (left) and cross-sectional SEM image (right) of the n–i–p perovskite/c-Si TSCs. **b** *J*–*V* characteristics of champion n–i–p perovskite/c-Si TSCs with traditional spin-coated and VTE-deposited spiro-OMeTAD. EQE spectra and 1–*R* curves of the perovskite (pink) and silicon (blue) sub-cells with **c** spin-coated and **d** VTE-deposited spiro-OMeTAD, where *R* is the reflectance of the devices. **e** Simulated *J*_ph_ plots of optical absorption and reflection with the spiro-OMeTAD thickness ranging from 5 to 400 nm. **f** Simulated *J*_ph_ mappings of optical absorption of TSCs, spiro-OMeTAD parasitic absorption, and reflection losses as functions of IZO and spiro-OMeTAD thicknesses. **g** *J*_SC_–*V*_OC_ × FF statistical graph of n–i–p perovskite/c-Si TSCs reported in the literature and this work. The related values are summarized in Table S5

We employ a mixed halide perovskite Cs_0.05_(MA_0.23_FA_0.77_)_0.95_Pb(Br_0.23_I_0.77_)_3_, with a bandgap energy (*E*_g_) of ~ 1.68 eV as the top-cell absorber, in conjunction with a SHJ solar cell as the bottom cell, to fabricate perovskite/c-Si TSCs [30]. The detailed device structure is illustrated in Fig. 1a, where the top subcell comprises a stack of an SnO_2_ ETL, a perovskite active layer, a spiro-OMeTAD HTL, a MoO_*x*_ buffer layer, an IZO transparent conductive electrode (TCE), an Ag grid, and an MgF_*x*_ anti-reflection coating (ARC). The experimental details for device fabrication are provided in Supplementary Information. The cross-sectional SEM image in Fig. 1a reveals a perovskite layer thickness of ~ 570 nm. To minimize parasitic absorption by spiro-OMeTAD, we employ VTE to precisely control its thickness to 10 nm. The temperature was controlled at approximately 350 °C to prevent thermal decomposition of spiro-OMeTAD, as determined by thermogravimetric analysis (Fig. S1). Notably, the introduction of a 2D perovskite layer to modify the surface of the polycrystalline 3D perovskite forms a 2D/3D perovskite heterojunction, enabling the use of an ultrathin and dopant-free spiro-OMeTAD HTL. This mechanism will be discussed in detail in subsequent sections.

The champion perovskite/c-Si TSC (0.135 cm^2^) with VTE-deposited spiro-OMeTAD achieves a high PCE of 29.73%, featuring a *V*_OC_ of 1.927 V, a fill factor (FF) of 80.80%, a *J*_SC_ of 19.09 mA cm^−2^, and a stabilized PCE of 29.38% (Table S1 and Fig. S2). The *J*–*V* curves of perovskite/c-Si TSCs with VTE-deposited and traditional spin-coated spiro-OMeTAD are present in Fig. 1b. Notably, the *J*_SC_ of VTE-based TSCs increases from 17.82 mA cm^−2^ (spin-coated TSC) to 19.09 mA cm^−2^. Additionally, the 1.012 cm^2^ area TSC achieves an efficiency of 28.77% (Fig. S3 and Table S2), with a certified PCE of 28.25% measured by a third-party-independent institution (Fig. S4). Figure S5 demonstrates that devices using MoOₓ alone as the hole transport layer perform poorly, confirming that spiro-OMeTAD is the dominant transport layer in the bilayer stack of spiro-OMeTAD and MoO*ₓ* [31, 32]. To evaluate the optical performance of TSCs, we provide the external quantum efficiency (EQE) spectra (Fig. 1c, d). For the VTE-deposited spiro-OMeTAD TSCs, the integrated currents of the perovskite top sub-cell and silicon bottom sub-cell are determined to be 19.10 and 19.01 mA cm^−2^, respectively, both surpassing those of the spin-coated devices (17.85 and 17.78 mA cm^−2^). The improved *J*_SC_ primarily results from reduced parasitic absorption by the ultrathin spiro-OMeTAD within the 300–400 nm wavelength range. Moreover, the spin-coated TSC exhibits a distinct valley-shaped EQE drop at wavelengths of 800–1000 nm, which is attributed to resonance reflection from the thick solution-processed spiro-OMeTAD layer (*i.e.*, 100–200 nm) [33]. Crucially, the light absorption of the perovskite did not decrease with the insertion of the 2D perovskite [34].

A high *J*_SC_ for n–i–p perovskite/c-Si TSCs using spiro-OMeTAD HTL can be achieved, provided that the spiro-OMeTAD thickness is carefully optimized, as further supported by numerical simulations (Fig. 1e, f) [28, 29]. The variations in the photogenerated current densities (*J*_ph_) of the sub-cells, parasitic absorption by spiro-OMeTAD, and total reflection as a function of the spiro-OMeTAD thickness are illustrated in Fig. 1e. Apparently, the parasitic absorption of spiro-OMeTAD decreases as its thickness is reduced, accompanied by lower reflection-induced current losses, thereby enhancing the overall current for the TSCs. Specifically, the *J*_ph_ losses due to reflection (parasitic absorption by spiro-OMeTAD) reduce from 4.32 (1.80) to 3.01 (0.16) mA cm^−2^ when reducing the spiro-OMeTAD thickness from 150 to 10 nm. The reduced reflection directly enhances the *J*_ph_ of the silicon bottom sub-cell by improving infrared photon absorption in the 800–1000 nm spectral range, where this sub-cell exhibits a dominant spectral response. To achieve a high PCE of n–i–p perovskite/c-Si TSCs, window layers with both low parasitic absorption and minimal thickness are critical for maximizing *J*_SC_. To further support this conclusion, the *J*_ph_ mappings under various thicknesses of the TCE and HTL layers (*i.e.*, IZO and spiro-OMeTAD) are present in Fig. 1f. Within the selected region of thin IZO (< 83 nm) and thin spiro-OMeTAD (< 52 nm), a high *J*_ph_ (> 19 mA cm^−2^) is observed for the TSCs, consistent with the reduced *J*_ph_ losses from spiro-OMeTAD and reflection. The *J*_ph_ mappings reveal that parasitic losses are primarily determined by the spiro-OMeTAD thickness, while reflection losses increase with thicker IZO or spiro-OMeTAD, leading to reduced optical performance of the TSCs, in agreement with the earlier analysis.

To gain a comprehensive understanding of the performance improvements of n–i–p perovskite/c-Si TSCs, we compare key parameters (*i.e.*, *J*_SC_ and *V*_OC_ × FF) from previous studies, as summarized in Fig. 1g. Compared to other works using spiro-OMeTAD as the HTL, the improved PCE in this study mainly stems from the higher *J*_SC_, as expected. Notably, the silicon bottom sub-cell in this work features a polished surface without intentional texturing. Nevertheless, to further enhance the performance of n–i–p perovskite/c-Si TSCs, particularly toward *J*_SC_ > 20 mA cm^−2^ and PCE > 30%, texturing the silicon bottom sub-cell is essential [2].

### VTE-Deposited Spiro-OMeTAD on 2D/3D Perovskite Heterojunction

**Fig. 2:**
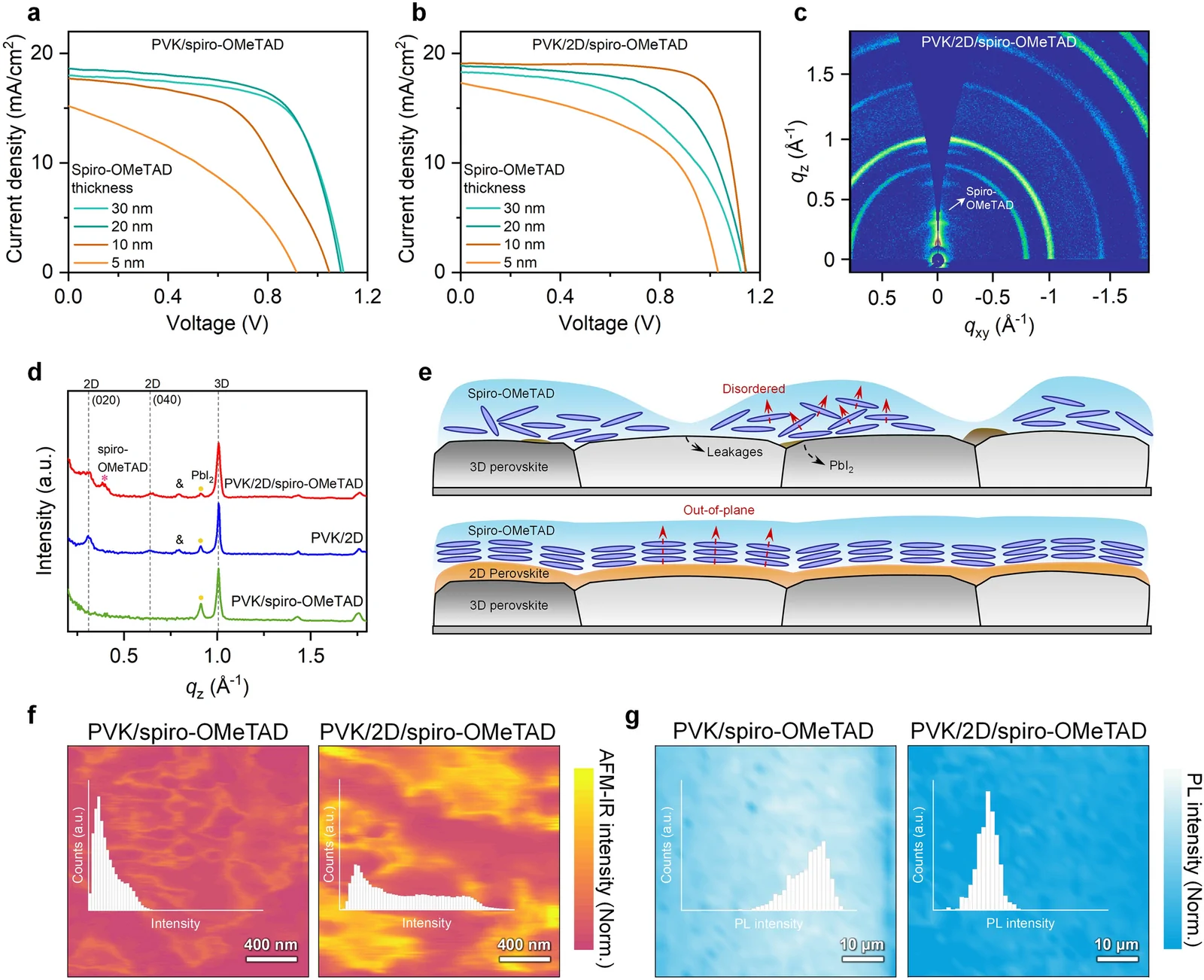
Electrical properties of the VTE-deposited spiro-OMeTAD on 2D/3D perovskite heterojunction. *J*–*V* curves of semitransparent perovskite solar cells **a** without and **b** with a 2D perovskite under various spiro-OMeTAD thicknesses. **c** GIWAXS patterns of the PVK/2D/HTL film. **d** Line profiles of GIWAXS patterns in Figs. 2c and S15 along the *q*_z_ axis, reflecting the out-of-plane orientations. **e** Schematic diagrams of spiro-OMeTAD arrangement on 3D perovskite and 2D/3D perovskite substrates (The spindle-shaped illustration does not represent the actual morphology of spiro-OMeTAD).** f** AFM-IR images corresponding 1505 cm^−1^ absorption, and **g** PL intensity mappings of the PVK/spiro-OMeTAD and PVK/2D/spiro-OMeTAD films. Insets show the corresponding statistical distributions of signal intensities

While a thin layer of spiro-OMeTAD is beneficial for n–i–p perovskite/c-Si TSCs to achieve high *J*_SC_, directly reducing its thickness can result in incomplete film coverage and potential current leakage, which may deteriorate the device electrical performance. Meanwhile, reducing or eliminating dopants can compromise the hole extraction capacity of spiro-OMeTAD [26, 35]. To address the insufficient charge-carrier transport in dopant-free spiro-OMeTAD, we introduce a 2D/3D perovskite heterojunction to enhance charge carrier extraction and selectivity. Initially, we explored a series of 2D passivation materials (BAI, PEAI, and TEAI) and systematically optimized concentrations of the best one (Fig. S6). We evaluate semitransparent perovskite solar cells with and without the 2D/3D heterojunction by reducing the thickness of VTE-deposited dopant-free spiro-OMeTAD from 30 to 5 nm (*J–V* curves in Fig. 2a, b), with the statistical parameters summarized in Figs. S7–S10 [36]. The introduction of the 2D/3D perovskite heterojunction alleviates the requirement for spiro-OMeTAD thickness while simultaneously increasing the shunt resistance. This finding is further supported by experimental results. Notably, dopant-free spiro-OMeTAD with a thickness exceeding 20 nm exhibits poor power output, likely due to its low electrical conductivity [35, 36]. Nevertheless, when the spiro-OMeTAD is reduced to below 10 nm, no PCE gain is observed, resulting in lower *V*_OC_ and FF [37, 38]. This could be attributed to poor interfacial contact in regions that are not fully capped by VTE-deposited spiro-OMeTAD. Consistently, the 10-nm-thick spiro-OMeTAD achieves the best PCE for the TSCs (Fig. S11).

To further confirm the influence of spiro-OMeTAD thickness on parasitic absorption, we examine the transmittance spectra based on spiro-OMeTAD/quartz glass structures (Fig. S12). The results show that a 5-nm-thick spiro-OMeTAD exhibits a transmittance deficit of only 8% at the wavelength of 400 nm. Even when the spiro-OMeTAD thickness increases to 30 nm, the parasitic absorption (up to 35% at 400 nm) remains significantly lower than the 95% observed in the traditional spin-coated spiro-OMeTAD (Fig. S12). The 10-nm spin-coated dopant-free spiro-OMeTAD and VTE-deposited spiro-OMeTAD layers exhibit similar transmittance, whereas the doped spin-coated spiro-OMeTAD shows markedly higher absorption. *J*_SC_ increment of VTE-deposited spiro-OMeTAD is mainly derived from the reduced thickness compared with the conventional spin-coated spiro-OMeTAD rather than the discard of dopants (Fig. S13). Additionally, we compare the parasitic absorption of a 10-nm-thick spiro-OMeTAD with that of VTE-deposited C_60_, a commonly used electron transport material in p–i–n TSCs, and find that the 10-nm-thick VTE-deposited spiro-OMeTAD exhibits lower parasitic absorption losses (Fig. S14).

To elucidate the interfacial coupling between the 2D perovskite and VTE-deposited spiro-OMeTAD, we conduct GIWAXS measurements using synchrotron radiation to obtain the scattering patterns of VTE-deposited spiro-OMeTAD. The high-flux X-ray beam enables quantitative analysis of molecular orientation anisotropy in the spiro-OMeTAD layer through azimuthal intensity distribution profiling, as systematically illustrated in Figs. 2c, d and S15 [39, 40]. Notably, two diffraction peaks at 0.31 and 0.64 Å^−1^ emerge after the post-treatment process on the original perovskite surface, corresponding to d-spacings of 20.2 and 9.8 Å, respectively. These values align with the lattice constants of (020) (*n* = 2) and (040) (*n* = 2) planes of (BA)_2_FAPb_2_Br_*x*_I_7–*x*_ 2D perovskite, indicating that the *n* = 2-dominated quasi-2D perovskite is prepared [41]. Additionally, a diffraction peak labeled as & at 0.79 Å^−1^ is observed, attributed to a gradual layer of the delta phase of the BA-related non-perovskite polymorph located within several tens of nanometers near the surface, which is beneficial for efficient charge transport by forming a funnel-like band alignment in the 2D/3D perovskite heterojunction [42]. An additional peak at 0.38 Å^−1^ is also identified, corresponding to the π–π stacking distance between spiro-OMeTAD molecules [39, 43]. These findings indicate a well-ordered molecular arrangement of spiro-OMeTAD and improved film coverage on the perovskite surface. This direct evidence suggests that the improved device performance is partially due to the reorganized spiro-OMeTAD accumulation on the 2D/3D perovskite heterojunction (Fig. 2e).

To confirm the local distribution of VTE-deposited spiro-OMeTAD with and without the 2D perovskite, atomic force microscopy-based infrared spectroscopy (AFM-IR) is employed (Fig. 2f). The AFM-IR signals are measured at 1505 cm^–1^, a characteristic IR absorption wavenumber of spiro-OMeTAD corresponding to the C − N stretching mode in its triphenylamine group [26]. The comparative analysis of AFM-IR and corresponding topographical signals (Fig. S16) of the PVK/spiro-OMeTAD and PVK/2D/spiro-OMeTAD films reveals significantly stronger AFM-IR intensity for the PVK/2D/spiro-OMeTAD film, confirming greater spiro-OMeTAD coverage on the perovskite surface. In contrast, the PVK/spiro-OMeTAD film exhibits weak AFM-IR signals in most areas, indicating insufficient capping and potential current leakage paths (Fig. 2e). Supporting this observation, a transmission electron microscopy (TEM) image (Fig. S17) demonstrates that spiro-OMeTAD forms a uniform and dense film conformal with the perovskite. To further validate the improved coverage of VTE-deposited spiro- OMeTAD, PL mapping measurements are conducted (Fig. 2g). Perovskite films are fabricated on bare glass substrates to exclude the influence of the ETL on the measurement results. PL mappings, conducted with a 2 × 2 µm^2^ scanning step and 60 × 60 µm^2^ area, reveal an irregular distribution for pristine perovskite, likely due to uneven PbI_2_ distribution, defect-rich grain boundaries, and surface roughness, in agreement with previous studies [44, 45]. When spiro-OMeTAD is deposited directly on perovskite, PL signals exhibit pronounced inhomogeneity: low-intensity (dark) regions are likely affected by charge-carrier extraction due to spiro-OMeTAD, while the high-intensity regions suggest incomplete coverage by spiro-OMeTAD. This spatial variation implies potential current leakage in ultrathin spiro-OMeTAD films. In contrast, the introduction of a 2D perovskite layer partially repairs grain boundaries and grains on the perovskite surface, as confirmed by the SEM results (Fig. S18). Meanwhile, AFM results reveal that the surface roughness of the 3D perovskite is significantly reduced from 25.0 to 15.5 nm after 2D perovskite modification, indicating a much smoother 3D perovskite surface (Fig. S19). The 2D interlayer molecules effectively passivate dangling bonds on the 3D perovskite and reduce surface irregularities during post-treatment [41].

### Electrical Performance of VTE-deposited Spiro-OMeTAD on 2D Perovskite

**Fig. 3:**
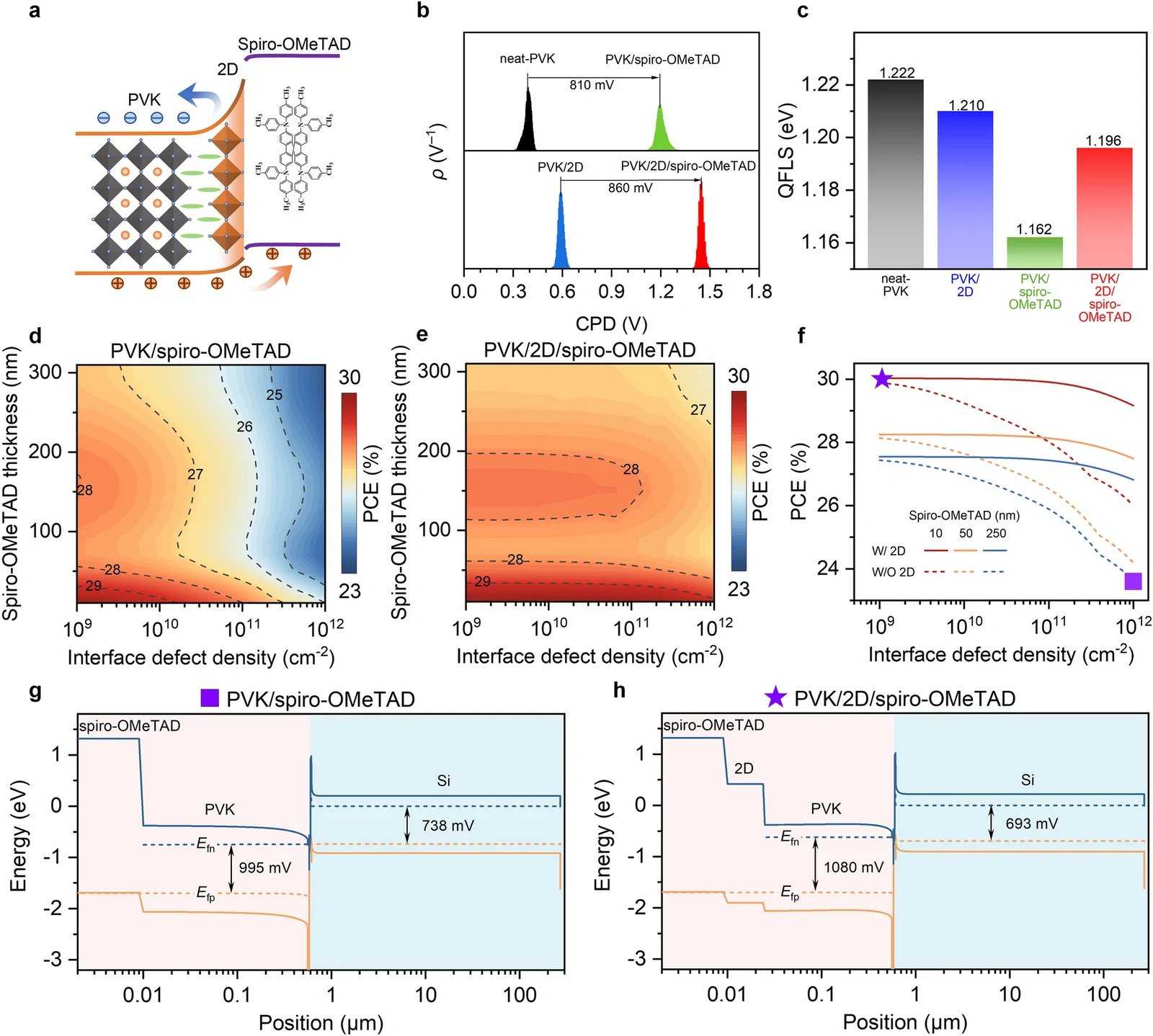
Energy band structures and interface defects on device performance**. a** Energy structure of a PVK/2D/spiro-OMeTAD stack. **b** Statistical distribution histograms of CPD obtained by KPFM. **c** QFLS values of the four related samples. Simulated PCEs of TSCs **d** without and **e** with a 2D perovskite as functions of the spiro-OMeTAD thickness and the defect density at the perovskite/spiro-OMeTAD interface. **f** Line plots of PCE values in Fig. 3d, e as a function of interface defect density for three typical spiro-OMeTAD thicknesses. Schematic diagrams of energy band structures of TSCs **g** without and **h** with the 2D perovskite layer at a fixed bias of 1.7 V (near the maximum power point)

To understand the electrical properties of VTE-deposited spiro-OMeTAD on 2D/3D perovskite, we conduct UPS and KPFM measurements. As revealed by the surface-sensitive UPS measurements, an upward band bending is induced by the 2D perovskite (Fig. 3a), consistent with previous results [41]. This energy offset signifies a deepening of the valence band maximum (VBM) of spiro-OMeTAD, indicating a reduced energy difference with the VBM of perovskite (Figs. S20-S22). This may be attributed to the improved arrangement of spiro-OMeTAD, as confirmed by GIWAXS results in Fig. 2b, further facilitating charge-carrier transport. The KPFM results support this conclusion (Figs. 3b and S23). The contact potential difference (CPD) between neat perovskite (neat-PVK) and 2D-capped perovskite (PVK/2D) is comparable, 0.39 and 0.59 V, respectively. After depositing spiro-OMeTAD, the average CPD values of PVK/spiro-OMeTAD and PVK/2D/spiro-OMeTAD samples are determined to be 1.20 and 1.45 V, respectively, indicating an increased work function. Furthermore, the half-peak width of the CPD distributions decreases from 203 to 122 mV, suggesting improved uniformity of spiro-OMeTAD coverage on perovskite films during vacuum evaporation, consistent with the PL mapping results. This conclusion is further supported by water contact angle measurements (Figs. S24 and S25). The hydrophilic perovskite surface becomes effectively encapsulated by the hydrophobic spiro-OMeTAD molecules (Fig. S26), resulting in significantly increasing the water contact angle, which has also been observed by other studies [46, 47].

To quantify the nonradiative losses of the 2D perovskite and VTE-deposited spiro-OMeTAD stacks, we conduct a quantitative comparison of the quasi-Fermi level splitting (QFLS) for the four related configurations (Fig. 3c) [48]. All samples are fabricated on bare glass substrates to independently analyze hole extraction performance. Compared with neat-PVK (1.222 eV), the QFLS reduction of PVK/2D (1.210 eV) indicates charge carrier separation by the 2D/3D perovskite heterojunction, in agreement with previous works [41, 42]. For the VTE-deposited spiro-OMeTAD sample, the QFLS is significantly quenched to 1.162 eV, as photogenerated holes are effectively transferred to the HTL, minimizing radiative recombination [49]. However, the QFLS of PVK/2D/spiro-OMeTAD (1.196 eV) is slightly stronger than that of PVK/spiro-OMeTAD, likely due to reduced interface defects (Fig. S27), which suppresses interfacial losses and enhances radiative recombination within the perovskite films. Additionally, 2D-based perovskite has minimal impact on the conductivity of VTE-deposited spiro-OMeTAD (Fig. S28).

To comprehensively evaluate the impact of the 2D/3D perovskite heterojunction on device performance, we investigate the PCEs of perovskite/c-Si TSCs with varying spiro-OMeTAD thicknesses and interfacial defect densities using the finite element method based on Silvaco TCAD software (Fig. 3d, e) [27]. For TSCs without the 2D/3D perovskite heterojunction, the contour lines in Fig. 3d demonstrate that the interface defect density has a more pronounced impact on PCE than the spiro-OMeTAD thickness. Achieving PCEs exceeding 29% requires both a thin spiro-OMeTAD (below 20 nm) and a low interface defect density (less than 10^10^ cm^–2^). In contrast, the introduction of a 2D perovskite layer significantly enhances defect tolerance, enabling PCEs above 29% even with defect densities ranging from 10^9^ to 10^12^ cm^–2^ for a 10-nm-thick spiro-OMeTAD layer. Figure 3f further illustrates the influence of the perovskite/HTL interface defect density on PCE. A higher defect density leads to a significant reduction in PCE, regardless of the presence of the 2D perovskite layer. This highlights that interface defects severely degrade PCE, irrespective of the spiro-OMeTAD thickness. Notably, the insensitivity of PCE to defects in the presence of the 2D perovskite aligns with experimental observations, indicating its ability to mitigate the dependence on HTL properties. The energy band diagrams at a fixed bias of 1.7 V (near the maximum power point, MPP) are shown for two typical cases (Fig. 3g, h), with dashed lines indicating the electron and hole Fermi levels. For the TSC without the 2D perovskite (with a spiro-OMeTAD thickness of 250 nm), the perovskite top sub-cell exhibits a moderate QFLS of 955 mV. This value increases to 1080 mV when the 2D/3D perovskite heterojunction is introduced (with a spiro-OMeTAD thickness of 10 nm). However, the QFLS of the silicon bottom sub-cell decreases from 738 to 693 mV, which is attributed to the fact that the silicon bottom sub-cells need to allocate a higher voltage when the perovskite top sub-cells are poor-performing [50]. This observation underscores the need for further optimization to balance current matching between the sub-cells at MPP conditions.

### Charge Carrier Dynamics of VTE-Deposited Spiro-OMeTAD on 2D Perovskite

**Fig. 4:**
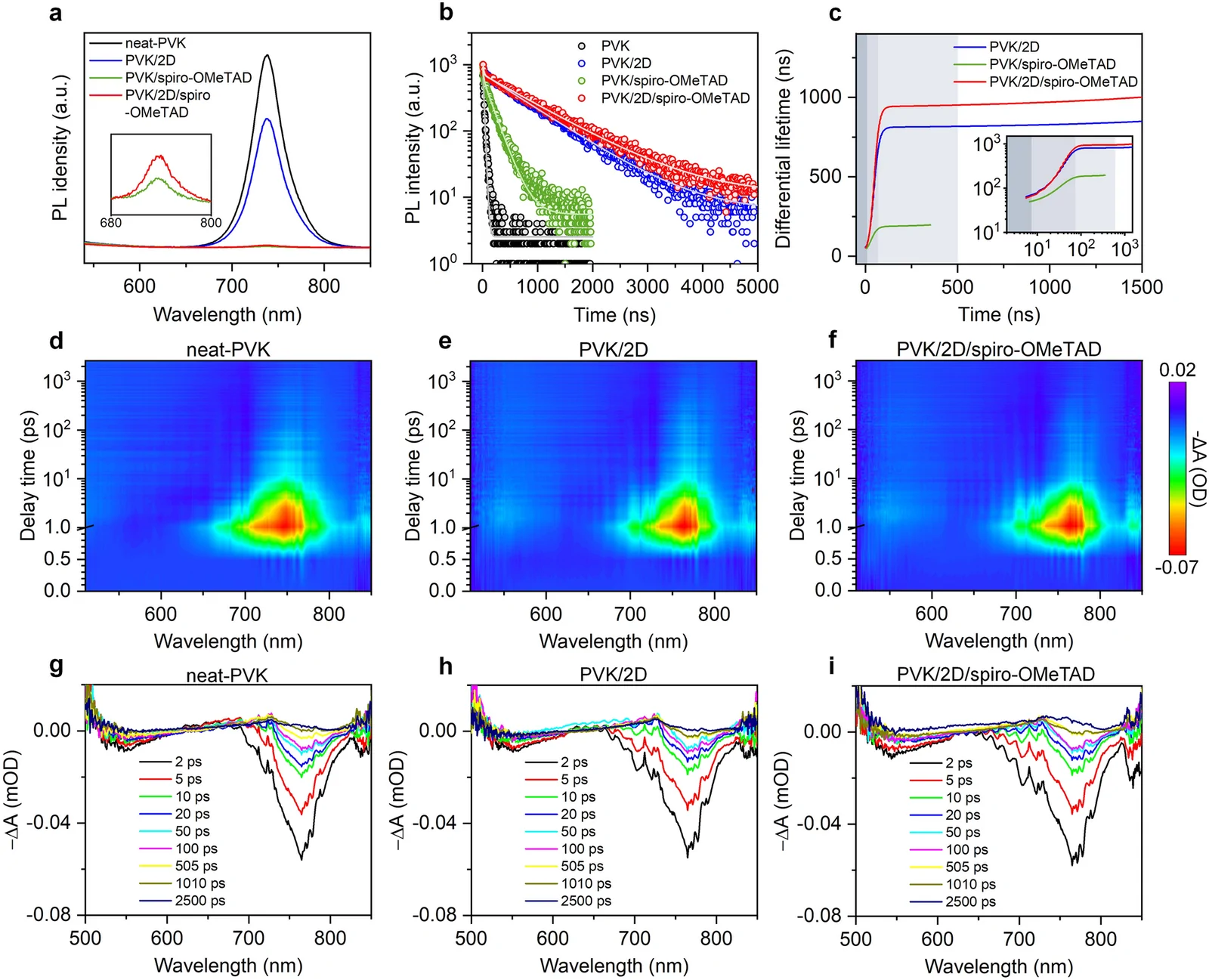
Electrical characterization of VTE-deposited spiro-OMeTAD on 2D perovskite. **a** Steady-state PL spectra, and **b** TrPL decay curves of the four related samples. **c** Computed differential lifetimes fitted from the transients in Fig. 4b. **d**-**f** Pseudocolor plots of TAS results, where the GSB signals are shown as functions of wavelengths and times. TAS evolutions of **g** neat-PVK, **h** PVK/2D and **i** PVK/2D/spiro-OMeTAD samples at various delay times

To gain deeper insights into charge-carrier dynamics at perovskite-related interfaces, we examine steady-state PL measurement on neat-PVK, PVK/2D, PVK/spiro-OMeTAD, and PVK/2D/spiro-OMeTAD samples. As shown in Fig. 4a, the PL spectra reveal that the deposition of a 2D perovskite layer on neat perovskite results in a reduced PL intensity. However, in samples incorporating the spiro-OMeTAD HTL, the introduction of the 2D perovskite leads to an enhancement in PL intensity, consistent with the QFLS results [47]. To explore the charge carrier dynamics, TrPL profiles are utilized to uncover various recombination and transport mechanisms, including trap-assisted recombination, radiative recombination, and charge transfer effects [51]. Figure 4b presents the PL decay curves of the four samples. The quenching of the PL signal in the neat perovskite is too rapid to clearly differentiate time constants, despite exhibiting the highest QFLS as extracted from photoluminescence quantum yield (PLQY). This observation may indicate a higher second-order recombination rate constant, leading to faster TrPL decay even under higher steady-state PL conditions [48]. In contrast, the TrPL curves for PVK/2D, PVK/spiro-OMeTAD, and PVK/2D/spiro-OMeTAD demonstrate a clear biexponential decay, indicating a charge transfer process with a short lifetime and a Shockley–Read–Hall recombination with a long lifetime [52].

To separate processes that reduce PL counts over time and directly extract the transient lifetimes at each time point, differential lifetimes are obtained by fitting the TrPL decay curves (Fig. 4c) [53, 54]. For both PVK/2D and PVK/2D/spiro-OMeTAD samples, the lifetimes asymptotically approach values exceeding 1.5 µs, suggesting minimized interfacial nonradiative recombination losses. The sharp rise at early times correlates with the charge transfer process, and the transition slopes from increasing lifetime to the plateau mark the end of charge transfer [53], which is influenced by charge transfer speed [39]. As shown in the inset of Fig. 4c, the saturation of the transition slopes starts at ~ 60 ns for the PVK/2D and PVK/2D/spiro-OMeTAD cases, slightly lower than the 75 ns observed for the PVK/spiro-OMeTAD reference, which is in agreement with the biexponential fitting of TrPL lifetimes in Table S3.

To further support the assertion that the charge carriers continuously transfer from the 3D perovskite to the 2D perovskite and spiro-OMeTAD, TAS measurements are carried out on the neat-PVK, PVK/2D, and PVK/2D/spiro-OMeTAD samples, as shown in the pseudocolor plots in Fig. 4d-f. The ground-state bleaching (GSB) of the neat-PVK is observed near the band edge region (Fig. 4g-i), corresponding to the population of excited charges and enabling analysis of charge transfer and recombination kinetics [27]. The GSB signals exhibit a bi-exponential decay starting at ~ 1 ps, with the short and long lifetimes attributed to charge carrier transport and trap-assisted recombination, respectively [55]. Notably, the samples with 2D perovskite modifications exhibit a shorter fast lifetime of 4.29 ps and a higher long lifetime of 288 ps compared to those of the only spiro-OMeTAD sample (4.81 and 176 ps, respectively). This indicates enhanced hole extraction and suppressed non-radiative recombination at the perovskite/spiro-OMeTAD interface (Fig. S29 and Table S4). Moreover, upon the introduction of 2D perovskite, a distinct peak emerges around 544 nm, appearing ~ 1 ps later than the 3D perovskite signals, directly confirming charge carrier transfer from the 3D perovskite to the 2D perovskite, which is in agreement with our earlier findings [41]. Compared with the 110-nm-doped spiro-OMeTAD, the ultrathin thickness of VTE-deposited spiro-OMeTAD reduces the demand for the conductivity of spiro-OMeTAD, and the introduction of 2D perovskite also improves the energy band matching ensuring efficient carrier transport.

### Stability of TSCs Featuring VTE-Deposited Spiro-OMeTAD

**Fig. 5:**
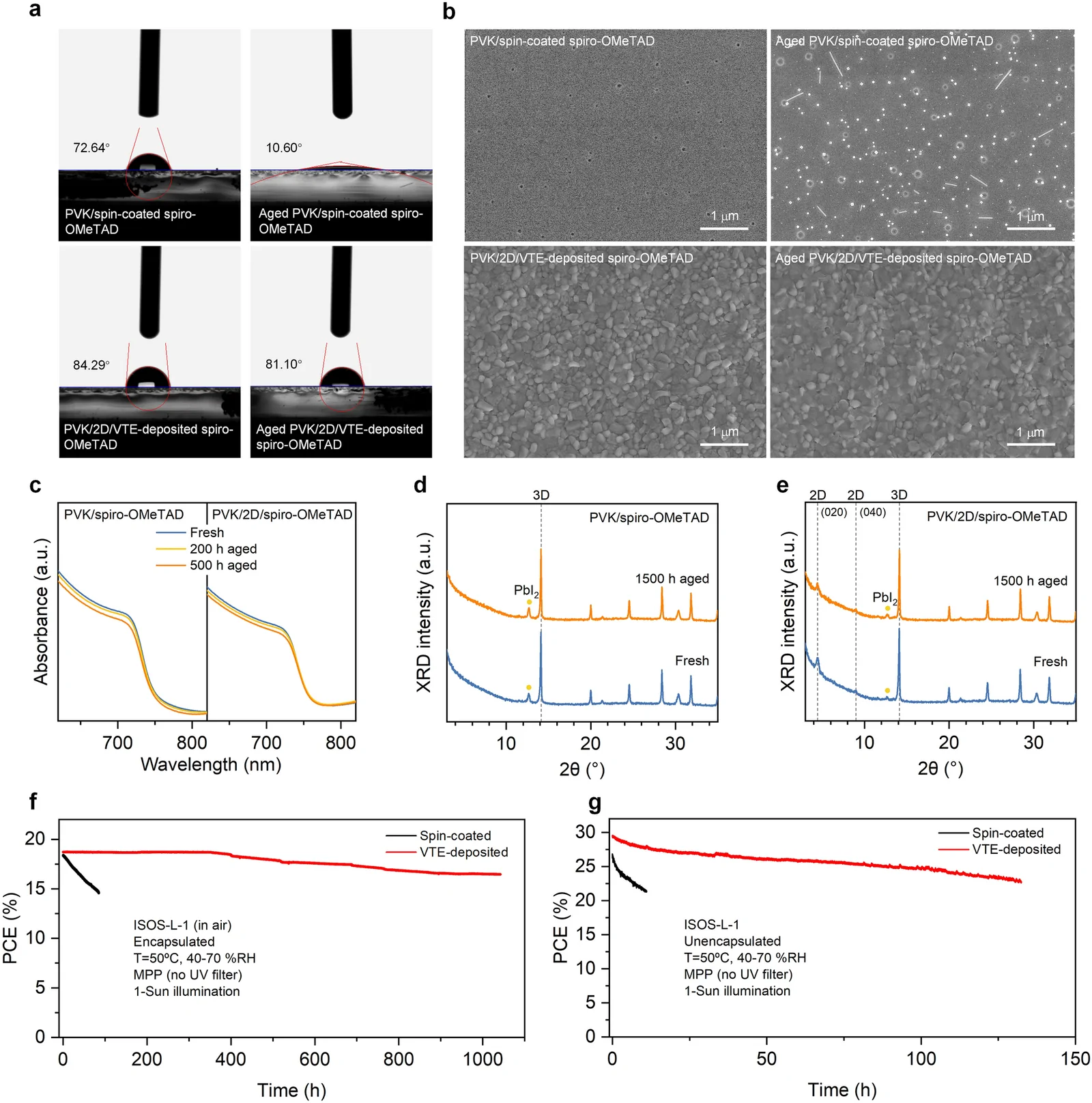
Stability measurements of TSCs featuring VTE-deposited spiro-OMeTAD.** a** Contact angle measurement of spin-coated spiro-OMeTAD and VTE-deposited undoped spiro-OMeTAD before and after aging in ambient air for 5 days. **b** SEM images of spin-coated spiro-OMeTAD and VTE-deposited spiro-OMeTAD deposited on PVK/SnO_2_/ITO/glass substrates before and after aging in air (humidity about 40%–70%) for 48 h. **c** Absorption spectra of perovskite/spiro-OMeTAD films with and without the 2D perovskite heterojunction (i.e., PVK/spiro-OMeTAD and PVK/2D/spiro-OMeTAD) under different aging times. **d** and **e** Tracking of XRD patterns from the two related cases. MPPT stability measurements of **f** the encapsulated semi-transparent perovskite solar cells and **g** the unencapsulated perovskite/c-Si TSCs in air

Comprehensive stability assessments are conducted on four representative structures to evaluate long-term perovskite degradation: neat-PVK, PVK/2D, PVK/spiro-OMeTAD, and PVK/2D/spiro-OMeTAD. Firstly, we perform contact angle measurements for the spin-coated and VTE-deposited spiro-OMeTAD on the perovskite (Fig. 5a). The VTE-deposited sample exhibits a contact angle of 84.29°, whereas the spin-coated sample shows a lower initial value of 72.64°. After 5 days of air exposure, the contact angle of the spin-coated sample drops sharply to 10.60°, while the VTE-deposited sample remains stable, with only a slight decrease of 3.19°–81.10°. Moreover, as shown in Fig. 5b, the surface morphology of the spin-coated spiro-OMeTAD film deteriorates rapidly upon air exposure due to Li salt crystallization and phase separation, whereas the dopant-free VTE-deposited spiro-OMeTAD retains its initial conformal morphology. This enhanced stability likely stems from the absence of hygroscopic and volatile dopants, which are typically required in spin-coated spiro-OMeTAD and known to induce hydrophilicity over time.

In addition, the optical absorbance degradation of the samples under accelerated aging conditions (~ 85 °C and AM 1.5G-equivalent illumination) is recorded (Figs. 5c and S30). Notably, with the presence of an additional 2D layer, PVK/2D exhibits a more pronounced absorbance reduction compared to neat-PVK, consistent with the findings of Huang et al. [56], which is attributed to the instability of BA⁺ cations in 2D layers when combined with FA⁺-based 3D perovskites under prolonged light exposure and high-temperature condition. However, comparative analysis reveals that inserting 2D perovskite between 3D perovskite and spiro-OMeTAD mitigates absorbance fading (Fig. 5c). This suggests that VTE-deposited spiro-OMeTAD on 2D/3D perovskite could partially suppress degradation pathways (Fig. S31). Additionally, phase stability is further evaluated through XRD patterns of the four related samples (Figs. 5d, e, and S32). In the as-deposited films, the diffraction peaks of PbI_2_ are notably lower in the samples with 2D perovskite, indicating the reduction in excess PbI_2_ due to its reaction with BA⁺ cations, which is beneficial for improving the stability of devices. After 1500 h aging in an ambient environment, 2D and 2D/spiro-OMeTAD-based samples exhibit clear diffraction peaks corresponding to typical 2D and 3D perovskite phases, while no significant increase in PbI₂ peaks is observed.

Furthermore, MPP tracking (MPPT) of semi-transparent single-junction perovskite solar cells and perovskite/c-Si TSCs with different HTLs is monitored under International Summit on Organic Photovoltaic Stability (ISOS)-L-1 condition (Fig. 5f, g) [57]. The initial PCE of tandem cells is 26.79% and 29.47% (18.40% and 18.73%) for the control and target tandem (single junction) device, respectively. The semi-transparent single-junction perovskite solar cell with VTE-deposited spiro-OMeTAD maintains 88% of its initial PCE after 1000 h, whereas the control device with spin-coated spiro-OMeTAD shows rapid degradation within 100 h. The unencapsulated perovskite/c-Si TSC shows a T80 (degradation period from 100% to 80%) of 110 h, which is 11 times more than the device with spin-coated spiro-OMeTAD. This further confirms that the combination of 2D perovskite and dopant-free VTE-deposited spiro-OMeTAD significantly improves the long-term stability of perovskite solar cells [58]. The single-junction perovskite solar cell shows negligible PCE loss in the first 350 h; however, the PCE of TSC decreases at the beginning of the test. We speculate that this result is related to the accumulation of photo-generated charge carriers by imperfect energy alignment of spiro-OMeTAD and perovskite, which is harmful to stability and easier to be observed in wide-*E*_g_ perovskite solar cells when illuminated from the HTL side in a tandem device [59]. Therefore, exploring new HTL materials with more matching energy alignment with wide-*E*_g_ perovskite is a key to further unlocking the stability and efficiency potential of perovskite-related TSCs, as our previous result attested [60]. Our results highlight the importance of eliminating hygroscopic and volatile components commonly found in traditional HTLs, such as spin-coated spiro-OMeTAD, which can significantly degrade device performance under prolonged exposure to environmental conditions.

## Conclusions

The PCE and stability of n–i–p perovskite/c-Si TSCs are limited by the commonly used HTL designs. This study proposes reducing the thickness of spiro-OMeTAD to 10 nm and eliminating dopants to fully unlock their performance potentials. This strategy can effectively suppress the parasitic absorption and reflecting losses of spiro-OMeTAD within the entire response spectrum of TSCs. The experimental results and optical simulations highlight that an HTL featuring ultrathin thickness and low parasitic absorption is crucial for high PCE n–i–p perovskite/c-Si TSCs. 2D/3D perovskite heterojunction is successfully used to make up the interface leakages due to the thickness reduction of HTL. It enables more even coverage and improved molecular arrangement of VTE-deposited spiro-OMeTAD. Electrical characterization and simulations reveal that the significantly improved tolerance to interface defects and effective passivation by 2D/3D perovskite heterojunction are the intrinsic mechanism of ultrathin and dopant-free spiro-OMeTAD in high efficiency TSCs. According to the long-term tracking under illuminations and heated conditions, it is found that the VTE-deposited spiro-OMeTAD can in turn alleviate the instability of 2D/3D perovskite heterojunction. For the single junction (tandem) device, the cell retains 88% (80%) of its initial PCE, after 1000 h (110 h) of continuous illumination under MPPT conditions. These results presented here provide an effective guideline for designing and optimizing efficient and stable n–i–p perovskite/c-Si solar cells.

It is important to note that the reported stability of the perovskite/c-Si TSCs featuring ultrathin spiro-OMeTAD presented in this study, with VTE-deposited MoO_x_ as the sputtered buffer layer, cannot currently be directly compared with that of the world-class p–i–n perovskite/c-Si TSCs. The contribution of a compact and conformal SnOx buffer layer by atomic layer deposition (ALD) to isolate moisture and oxidize gases can enhance the stability of p–i–n perovskite/c-Si TSCs. Additionally, the use of one-side polished SHJ solar cells to prepare the perovskite/c-Si TSCs in this work may limit the* J*_SC_ due to the absence of effective light-trapping textures. However, for proof-of-concept purposes, these limitations do not overturn the conclusions demonstrated. In summary, the results displayed in this work provide valuable insights into minimizing optical losses of HTL in n–i–p tandems, contributing to a deeper understanding of the structure design and promoting perovskite/c-Si TSCs toward higher stability and efficiency.

To further reduce the PCE gaps and approach the ~ 34% of p–i–n tandems, future efforts could be concerted: (i) The integration of VTE-deposited spiro-OMeTAD with appropriate ETL fabrications on textured substrates by employing vacuum-depositing method is expected to bring n–i–p perovskite/c-Si TSCs closer to and potentially surpass p–i–n TSCs. (ii) Most critically, improving the *V*_OC_ of wide bandgap n–i–p PSCs by suppressed recombination and improved charge extraction through the development of novel transport materials.

## Supplementary Information

Below is the link to the electronic supplementary material.

Supplementary file1 (DOCX 24472 KB)
