# Supplementary material for: Dopant-Free Ultra-Thin Spiro-OMeTAD Enables Near 30%-Efficient n–i–p Perovskite/Silicon Tandem Solar Cells
Source: Nanomicro Lett. 2026 Jan 14;18:116. doi: 10.1007/s40820-025-01962-3 (PMC12799825; doi:10.1007/s40820-025-01962-3)
Supplement: Supplementary file 1 — Supplementary file1 (DOCX 24472 KB) [file 40820_2025_1962_MOESM1_ESM.docx]

Supporting Information for

**Dopant-Free Ultra-Thin Spiro-OMeTAD Enables Near 30%-Efficient**

**n–i–p Perovskite/Silicon Tandem Solar Cells**

Xiangying Xue^1, #^, Weichuang Yang^1, #,^ *, Zhiqin Ying^1^, Fangfang Cao^1^, Yuheng Zeng^1^, Zhenhai Yang^3, 1, *^, Xi Yang^1, 2,^ * and Jichun Ye^1, 2,^ *

^1^ Zhejiang Provincial Engineering Research Center of Energy Optoelectronic Materials and Devices, Ningbo Institute of Materials Technology and Engineering, Chinese Academy of Sciences (CAS), Ningbo 315201, P. R. China

^2^ Research Center for Wide Bandgap Semiconductors and Devices, YongJiang Laboratory, Ningbo 315202, P. R. China

^3^ School of Optoelectronic Science and Engineering & Collaborative Innovation Center of Suzhou Nano Science and Technology; Key Lab of Advanced Optical Manufacturing Technologies of Jiangsu Province & Key Lab of Modern Optical Technologies of Education Ministry of China, Soochow University, Suzhou 215006, P. R. China

^#^ Xiangying Xue and Weichuang Yang contributed equally to this work.

*Corresponding authors. E-mail: [yangweichuang@nimte.ac.cn](mailto:yangweichuang@nimte.ac.cn) (Weichuang Yang); [zhyang@suda.edu.cn](mailto:zhyang@suda.edu.cn) (Zhenhai Yang); [yangx@nimte.ac.cn](mailto:yangx@nimte.ac.cn) (Xi Yang); [jichun.ye@nimte.ac.cn](mailto:jichun.ye@nimte.ac.cn) (Jichun Ye)

**Supplementary Note S1**

Quasi-Fermi Level Splitting (QFLS) characterizes the disparity in density of free photogenerated electrons and holes between the conduction and valence bands in a solar cell [S1-S3]. Direct determination of QFLS is achievable through absolute Photoluminescence (PL) measurements, assuming that all PL arises from radiative recombination of free charges in the perovskite (PVK).

The Photoluminescence Quantum Yield (PLQY) is defined as the ratio of emitted photon flux (*φ*_E_) resulting from free carrier recombination in the PVK to the absorbed photon flux (*φ*_A_). Equivalently, it is the ratio of the total radiative recombination current (*J*_rad_) to the generation current (*J*_G_). Under open-circuit voltage (*V*_oc_) conditions, where the net current is zero, *J*_G_ equals the recombination current (*J*_R_), encompassing the radiative (*J*_rad_) and all non-radiative recombination processes (*J*_non-rad_) in the PVK and other layers/interfaces.

$PLQY=\frac{\varphi_{E}}{\varphi_{A}}=\frac{J_{rad}}{J_{G}}=\frac{J_{rad}}{J_{R}}=\frac{J_{rad}}{J_{rad}+J_{non-rad}}$ (S1)

In quasi-equilibrium under steady-state illumination, the density of free carriers in the valence and conduction bands of the PVK is related to QFLS by the equation:

$n^{2}=N_{c}N_{v}\cdot e^{(E_{c}-E_{v})/(k_{B}T)}\cdot e^{(E_{F,e}-E_{F,h})/(k_{B}T)}=n_{i}^{2}\cdot e^{(QFLS)/(k_{B}T)}$ (S2)

Here, *N*_c_ and *N*_v_ are the effective density of states in the conduction and valence bands, *T* is the temperature, *k*_b_ is the Boltzmann constant, *E*_c_ and *E*_v_ are the conduction and valence band energy levels, *n*_i_ is the intrinsic carrier density in the dark, and QFLS represents the splitting of the quasi-Fermi levels.

Relating this to the radiative recombination current (*J*_rad_) and the dark radiative recombination current (*J*_0, rad_), we get:

$J_{rad}=J_{0,rad}\cdot e^{(QFLS)/(k_{B}T)}$ (S3)

Combining this with Equation (1), we have:

$QFLS=k_{B}T\cdot\ln(PLQY\frac{J_{G}}{J_{0,rad}})=k_{B}T\cdot\ln(PLQY)+QFLS_{rad}$ (S4)

Here, QFLS_rad_ is the radiative limit of the semiconductor material, representing the maximum achievable splitting of the quasi-Fermi levels (hence the *V*_oc_) in the absence of nonradiative recombination.

**Supplementary** **Figures and Tables**

**Fig. S1** Thermal gravimetric analysis (TGA) of the spiro-OMeTAD powders. To ascertain an appropriate temperature setting for the quartz crucible during vacuum thermal evaporation and prevent thermal degradation of spiro-OMeTAD, TGA measurement is used to acquire its decomposition point. The temperature align features a typical decomposition temperature of small organic molecules, showing apparent weight losses at ~400°C, which is in agreement with previous report [S4]. Therefore, spiro-OMeTAD is evaporated at a monitored temperature of ~350°C, achieved by a ramp-up of 20°C/min, a deposition rate of 0.1 Å/s measured by quartz crystal monitors, and a substrate holder without intentional heat control.

**Fig. S2** 10 min stabilized power output (SPO) PCE of the champion perovskite/c-Si TSCs with evaporated spiro-OMeTAD

**Fig. S3** *J-V* characteristics of n–i–p perovskite/c-Si TSCs for 1.01 cm^2^ with traditional spin-coated and VTE-deposited spiro-OMeTAD


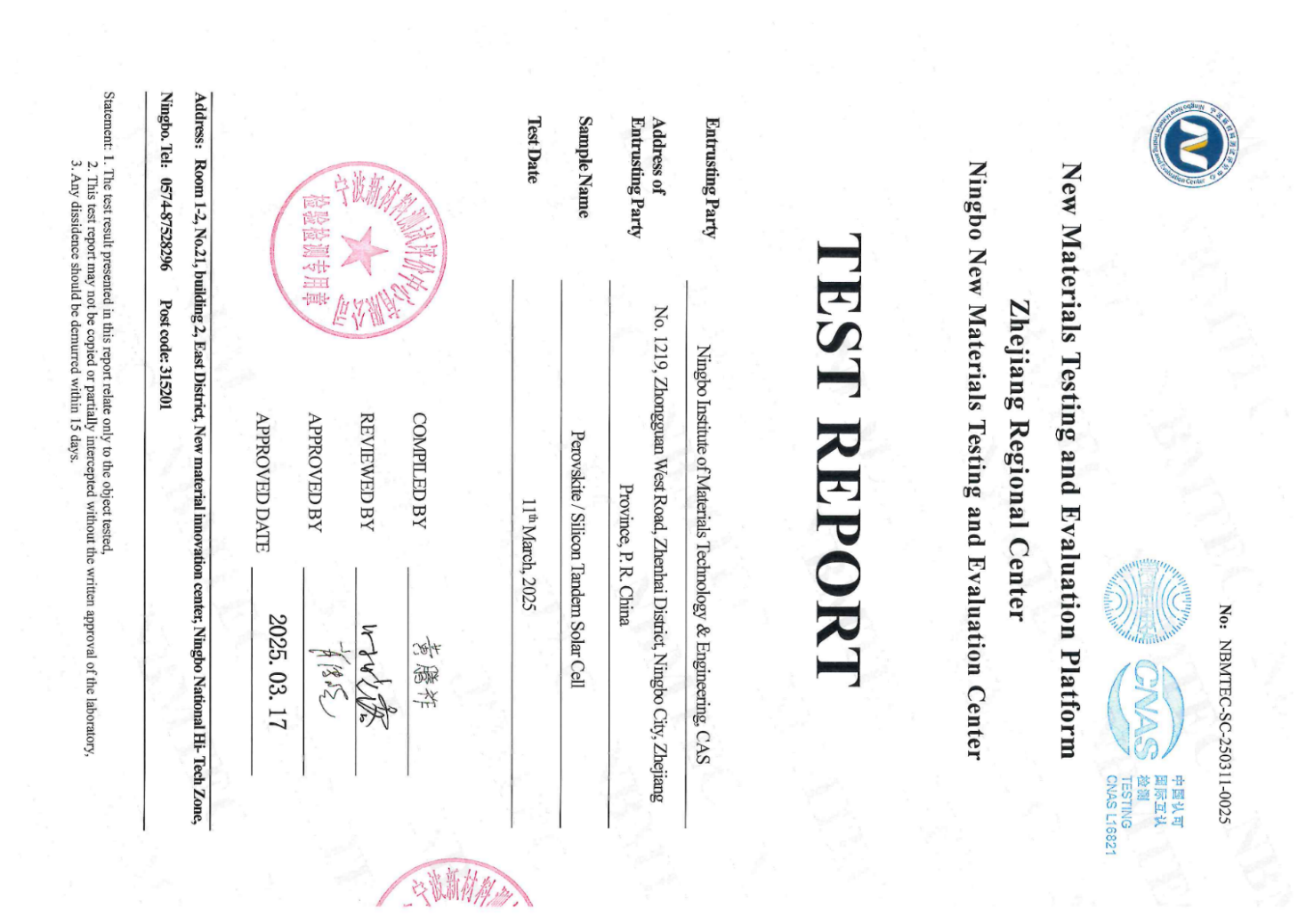

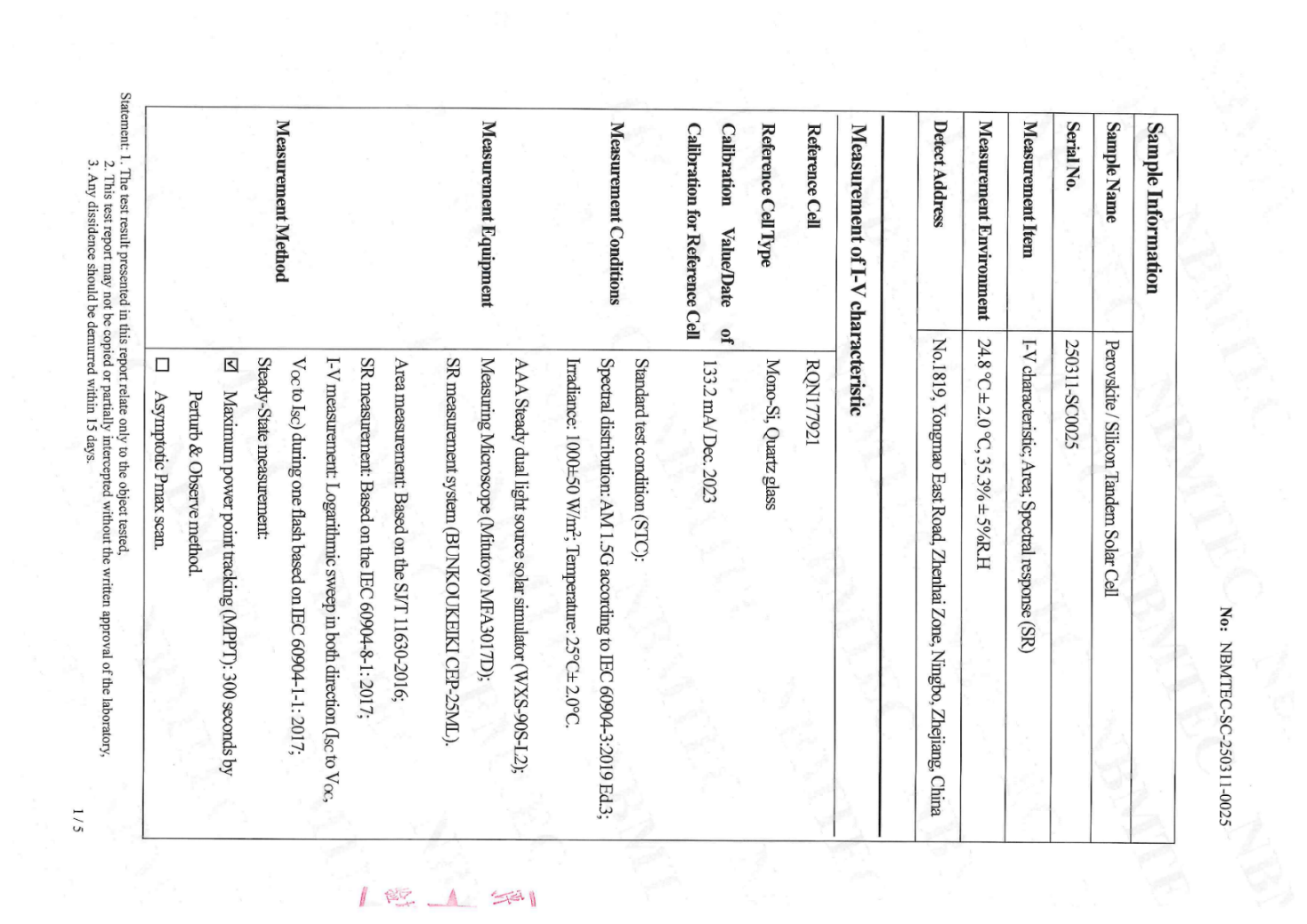

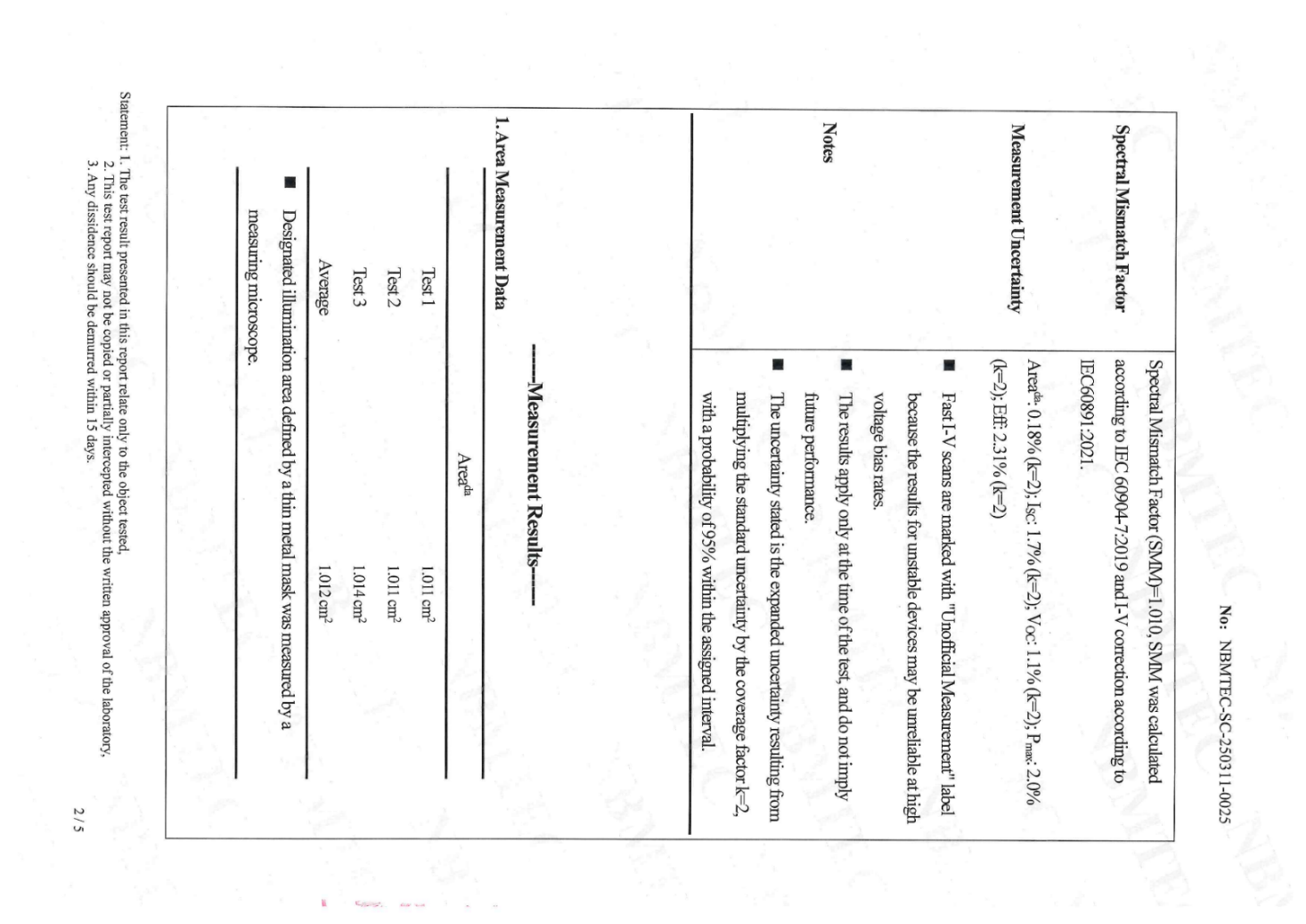

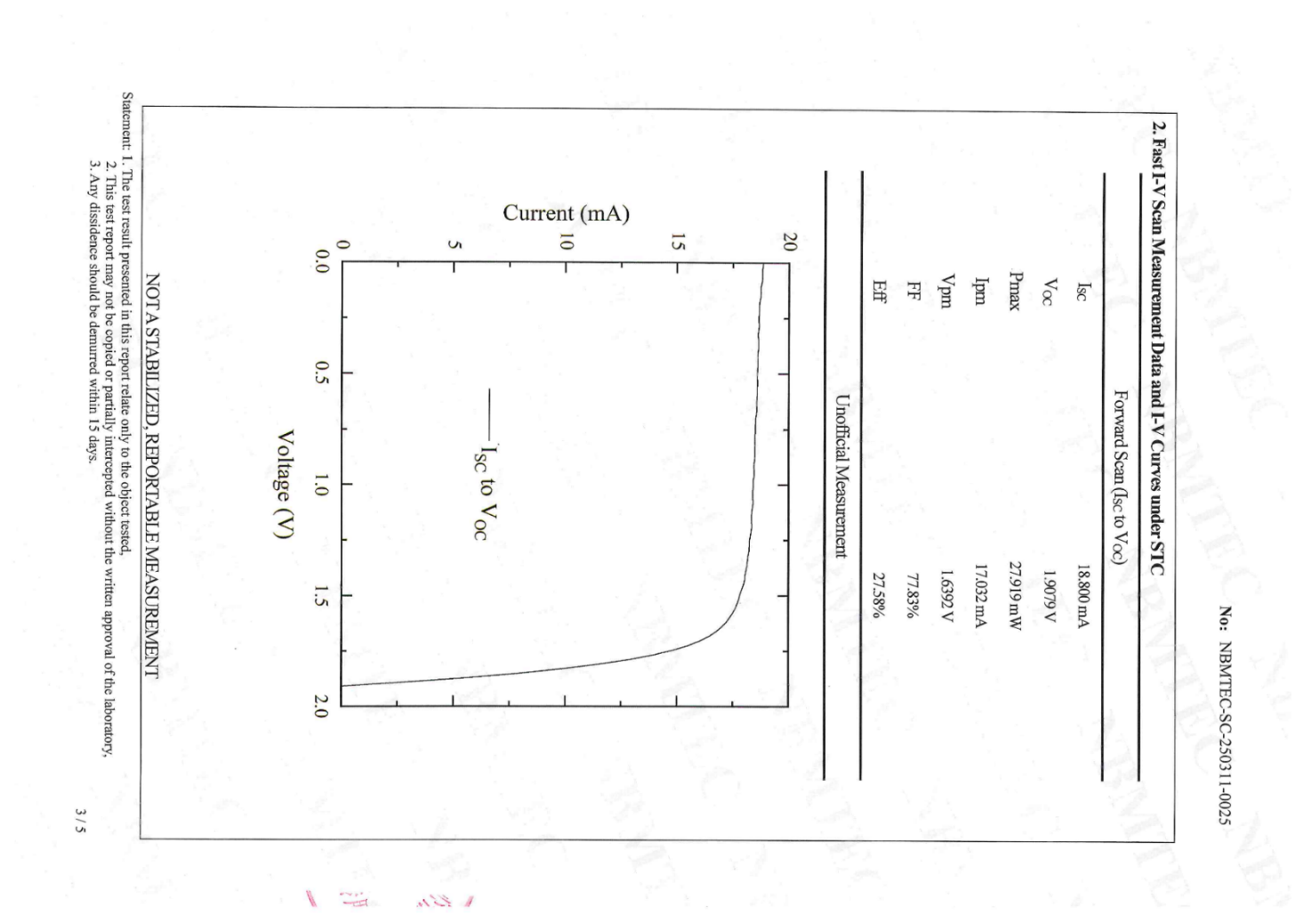

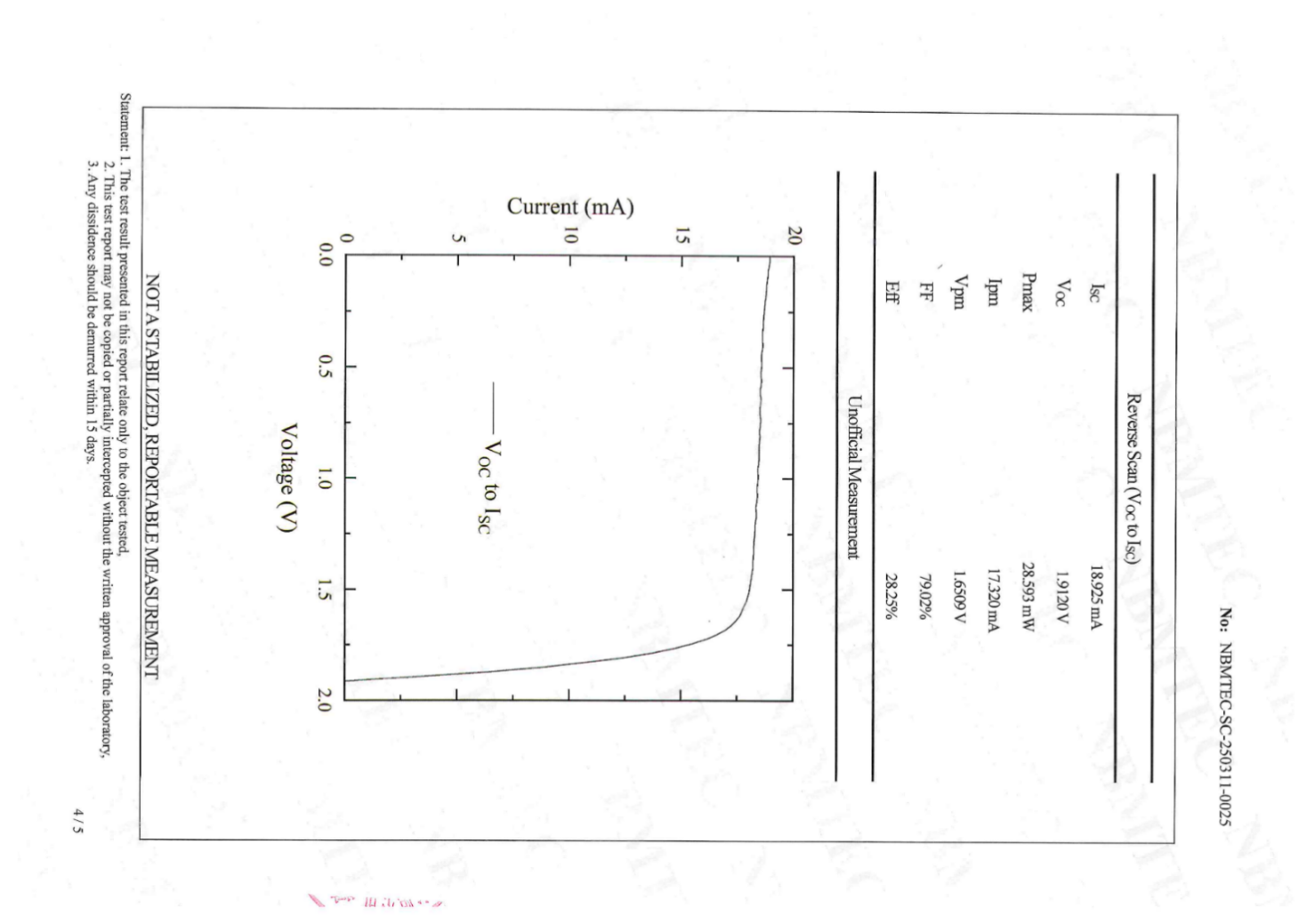

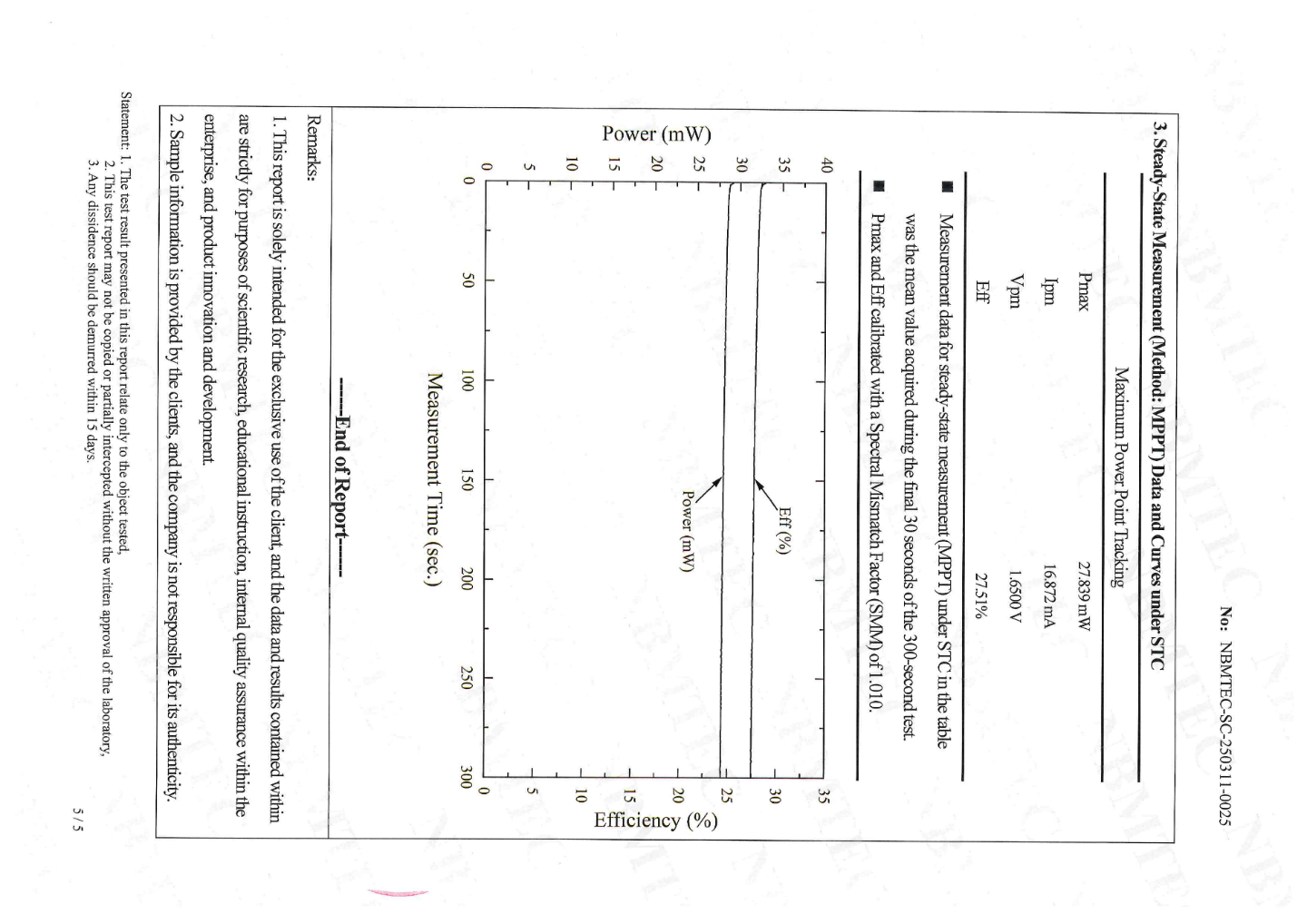


**Fig. S4** Independent efficiency measurement of TSC using dual-source solar simulator at New Materials Testing and Evaluation Platform Zhejiang Regional Center. The effective area is defined by an opaque mask with a 1.012 cm^2^ aperture area. The *V*_OC_ is 1.9120 V, *I*_SC_ is 18.925 mA, FF is 79.02% and PCE is 28.25% in the reverse voltage swept (*V*_OC_→*J*_SC_) *I-V* curve, and the *V*_OC_ is 1.9079 V, *I*_SC_ is 18.800 mA/cm^2^, FF is 77.83% and PCE is 27.58% in the forward voltage swept (*J*_SC_→*V*_OC_) *I-V* curve. The stable PCE is 27.51% by a steady-state measurement (Method: MPPT) of 300s

**Fig. S5** *J-V* curves of n–i–p PSTSCs with MoO_x_ and without spiro-OMeTAD. Here, we fabricate spiro-OMeTAD-free tandems with a structure of Si sub-cell/ITO/SnO_2_/Perovskite/MoO_x_/ITO/Ag. The *J-V* curves are presented in the Fig. S5, with a *J*_SC_ of 18.76 mA/cm^2^, a *V*_OC_ of 1.76 V, an FF of 52.68%, and a PCE of 17.39%. A modest *J*_SC_ indicates that removing the HTL can indeed achieve a decent light absorption of tandems. However, the low *V*_OC_, FF, and PCE demonstrates that direct contacting MoOₓ to perovskite is unable to effectively realize carrier extraction. Hence, the MoO_x_ in this study is work more as a buffer layer rather than an HTL

**Fig. S6** *J*-*V* curves of cells **a** with different 2D materials (BAI PEAI TEAI) and **b** with different concentration of BAI. By comparing the different 2D passivation agents, we found that devices passivated with BAI delivered the highest efficiencies. Further optimization of BAI concentration revealed that 2 mg/ml yields the highest fill factor and the best overall performance, indicating that 2 mg/ml BAI passivation pairs optimally with the 10 nm VTE-deposited spiro-OMeTAD

**Fig. S7** The statistical forward-scan data of semi-transparent perovskite solar cells without 2D perovskite, including **a** *J*_SC_, **b** *V*_OC_, **c** FF and **d** PCE versus evaporated spiro-OMeTAD thicknesses, extracted from the *J*-*V* measurements by illuminating from the hole-transporting layer (HTL) side

**Fig. S8** The statistical reverse-scan data of semi-transparent perovskite solar cells without 2D perovskite, including **a** *J*_SC_, **b** *V*_OC_, **c** FF and **d** PCE versus evaporated spiro-OMeTAD thicknesses, extracted from the *J-V* measurements by illuminating from the hole-transporting layer (HTL) side. It is evident that directly using evaporated undoped spiro-OMeTAD as the HTL just yields modest efficiency, which has been reported in the previous study [5]. Notably, the PCEs increase with spiro-OMeTAD thickness when the spiro-OMeTAD layer is less than 20 nm. This phenomenon may be attributed to the leakage regions that are not well covered by the island growth spiro-OMeTAD. For thicker situations, *i.e.*, when HTL thickness is 30 nm, the FFs and *V*_OC_s increase due to improved contact within HTL and perovskite, while the increased parasitic absorption in HTL results in lower *J*_SC._

**Fig. S9** The statistical forward-scan data of semi-transparent perovskite solar cells with 2D perovskite, including **a** *J*_SC_, **b** *V*_OC_, **c** FF and **d** PCE versus evaporated spiro-OMeTAD thicknesses, extracted from the *J*-*V* measurements by illuminating from the hole-transporting layer (HTL) side

**Fig. S10** The statistical reverse-scan data of semi-transparent perovskite solar cells with 2D perovskite, including **a** *J*_SC_, **b** *V*_OC_, **c** FF and **d** PCE of semi-transparent perovskite solar cells with 2D perovskite versus evaporated spiro-OMeTAD thicknesses, extracted from the *J-V* measurements by illuminating from the hole-transporting layer (HTL) side. The champion cell emerges in the 10 nm condition, whereas below this thickness, the cells exhibit relatively lower FFs, which could be caused by the leakages. As the thickness exceeds 10 nm, the PCEs constantly reduce due to the contracted FFs along with stronger parasitic absorption and lower *J*_SC_s. The trends in tandem cells are similar to these results as follows in Figure S7.

**Fig. S11** *J-V* characteristics of perovskite/c-Si TSCs with different thicknesses of evaporated spiro-OMeTAD. In the presence of a 2D perovskite, a 10 nm-thick spiro-OMeTAD is sufficient for efficient hole extraction, which is confirmed by the HTL coverage measurement as scanning electron microscopy (SEM) results in Figure S10. When the thickness exceeds 10 nm, the series resistance increases significantly, along with higher parasitic absorption, resulting in a reduction in FFs, *J*_SC_, and PCE.

**Fig. S12** Transmittance spectra of evaporated spiro-OMeTAD with different thicknesses

**Fig. S13** Transmittance spectra of 10 nm dopant-free spin-coated spiro-OMeTAD, doped spiro-OMeTAD, and dopant-free VTE-deposited spiro-OMeTAD. Using a profilometer, we confirmed that the undoped spin-coated spiro-OMeTAD layer was ≈ 10 nm thick, and we prepared the doped solution at the same spiro-OMeTAD concentration. The transmission spectrum of the 10 nm undoped spiro-OMeTAD closely matches that of the VTE-deposited film, whereas the transmittance drops noticeably after doping. This decrease arises because the addition of TBP markedly improves the film-forming ability of spiro-OMeTAD, and subsequent lithium-salt doping oxidizes the spiro-OMeTAD.

**Fig. S14** Transmittance spectra of target HTL and commonly-used window layers

To comprehensively evaluate the light absorption characteristics of the ultra-thin deposited spiro-OMeTAD in various commonly-used window layers in perovskite solar cells horizontally, demonstrating its parasitic absorption, we deposited spiro-OMeTAD doped with Li salt, cobalt salt, and tBP on white glass using spin coating. In Figure S7, the spin-coated Li-doped spiro-OMeTAD exhibits strong absorption in the range of 300-400 nm, attributed to the inherent absorption properties of spiro-OMeTAD. Correspondingly, the transmitted curve of the 10 nm-thick spiro-OMeTAD film also shows absorption peaks at these wavelengths. However, with reduced thickness, the transmittance of the 10 nm-thick evaporated spiro-OMeTAD is 88.6% at 400 nm, higher than that of the spin-coated Li-doped spiro-OMeTAD, which exhibits absorption not only between 300–400 nm but also in the longer wavelength (> 800 nm). Meanwhile, the transmittance of evaporated spiro-OMeTAD is higher than C60 ETL with a 20 nm thickness that is commonly used in p–i–n perovskite/c-Si TSCs.

**Fig. S15** GIWAXS patterns of **a** PVK/VTE-deposited spiro-OMeTAD and **b** PVK/2D/VTE-deposited spiro-OMeTAD samples

**Fig. S16** Topography of the measured area in AFM-IR characteristic. This result reveals that the surface of the PVK/2D/spiro-OMeTAD is smoother than that of the PVK/spiro-OMeTAD film, with the roughness reducing from 11.06 nm to 4.8 nm, which is advantages for uniform deposition of the VTE spiro-OMeTAD on the perovskite surface.

**Fig. S17 a** Depth profiles of different elements obtained from TEM. **b** High-angle annular dark-field image in TEM and the corresponding EDXS mappings. TEM measurements were employed to evaluate the conformality of the spiro-OMeTAD layer. The structure of the test sample was TOPCon silicon bottom cell/ITO/SnOx/perovskite/2D-perovskite/spiro-OMeTAD/MoOx/IZO. The energy-dispersive X-ray spectroscopy (EDXS) analysis also illustrates this structure. A distinct C signal can be observed between the Pb and Mo signals, indicating the presence of spiro-OMeTAD at the perovskite/MoOx interface. The ultra-thin nature (~10 nm) and uniform coverage of the spiro-OMeTAD layer can be confirmed by TEM results.

**Fig. S18** SEM images of (**a**) neat-PVK, (**b**) PVK/2D, (**c**) PVK/HTL, and (**d**) PVK/2D/HTL

On the clean ITO substrate, we spin-coated tin oxide and perovskite as the control group. Subsequently, we deposited 2D perovskite, spiro-OMeTAD, and 2D/spiro-OMeTAD as the target groups. SEM was employed to compare the surface morphology. The surface of the control sample exhibits a typical 3D perovskite morphology with closely packed polycrystalline perovskite grains. In Figure S9b, after treating the surface of the 3D perovskite with a precursor solution of 2D perovskite, the 2D perovskite covers the surface perovskite grains and grain boundaries, resulting in a more uniform and smooth appearance. After depositing a 10 nm-thick spiro-OMeTAD, the surface of the sample shows no significant change, indicating that the HTL we fabricated is thin and conformal with the underlying perovskite film. Based on this, to achieve better deposition of the upper film, the fabrication of the 2D perovskite layer is essential. A smoother perovskite layer not only allows the upper HTL to be more uniform and denser but also, due to the thinness of the HTL, conforms with the surface of the perovskite layer. The roughness of the perovskite layer directly affects the deposition of the upper layers, including molybdenum oxide, IZO, and the electrode. Importantly, the 2D layer completely covers the grain boundaries of the 3D perovskite, preventing molecules from the deposited upper layers from penetrating through the perovskite layer. This barrier effect enhances device stability by preventing undesirable doping.

**Fig. S19** The AFM measurement of pre- (left) and post-treated (right) samples by BAI solution and thermal annealing. To compare the surface modifications of 3D perovskite after 2D post-treatment, we conducted atomic force microscopy (AFM) on both pre- and post-treated samples, as shown in Figure R2. The average roughness decreased from 25.0 nm to 15.5 nm, demonstrating that 2D perovskite facilitates more uniform deposition of spiro-OMeTAD despite both the 3D perovskite and BAI-based 2D perovskite and possessing similar functional groups.

**Fig. S20** UPS spectra around the cutoff and onset energy ranges. The calculated HOMO levels for neat-PVK, PVK/2D, PVK/HTL, and PVK/2D/HTL samples are –5.90 eV, –5.74 eV, –5.03 eV and –5.14 eV, respectively

**Fig. S21** UPS results of **a**) neat-PVK, **b**) PVK/2D, **c**) PVK/2D/VTE-deposited spiro-OMeTAD, and d) PVK/VTE-deposited spiro-OMeTAD

**Fig. S22** Energy levels extracted from UPS measurements in Fig. S14

**Fig. S23** KPFM images of neat-PVK, PVK/2D, PVK/spiro-OMeTAD, and PVK/2D/ spiro-OMeTAD samples. The lower row displays the cross-section lines of the potential maps taken following the indicator lines above each image

**Fig. S24** Contact angle measurements by dropping water droplets on surface of neat-PVK, PVK/2D, PVK/spiro-OMeTAD and PVK/2D/spiro-OMeTAD samples. The contact angle of the neat perovskite layer is 52.23° and improved to 64.71° after capped by 2D perovskite. A water contact angle of 86.57° is recorded as Figure R7, which is similar to that of the PVK/2D/10 nm VTE-deposited spiro-OMeTAD (84.29°) and higher than the PVK/10 nm VTE-deposited spiro-OMeTAD (69.31°), indicating that the 2D layer can effectively improve the coverage of spiro-OMeTAD on perovskite.

**Fig. S25** Statistical graph of contact angles. Three points are measured for each case


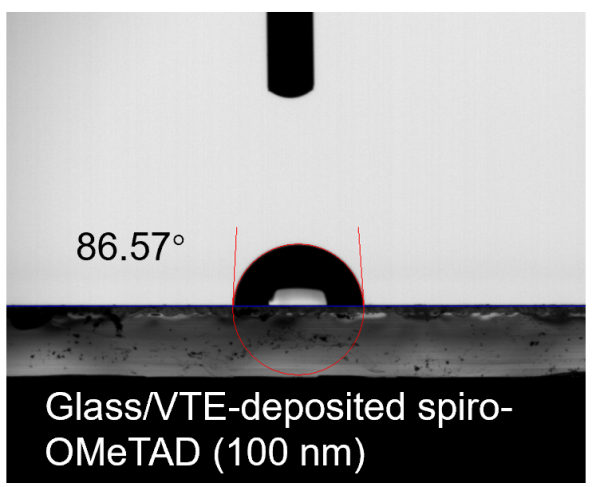


**Fig. S26** Spiro-OMeTAD deposited directly onto glass by vacuum thermal evaporation exhibits a contact angle of 86.57°, indicating its hydrophobic property

**Fig. S27** *I-V* curves of the space-charge-limited current (SCLC) measurements for the HTL-only devices, using structures glass/ITO/MeO-2PACz/perovskite/evaporated spiro-OMeTAD/Au The defect density can be calculated according to the following equation:

$$N_{t}=\frac{2\varepsilon\varepsilon_{0}V_{TFL}}{eL^{2}}$$

where *ε* and *ε*_0_ are the dielectric constant of the perovskite and the vacuum permittivity, respectively, *V*_TFL_ is the trap-filled limit voltage, *L* is the thickness of the perovskite film, and *e* is the elementary charge [S6]. According to this equation, the *N*_t_ of the sample with 2D perovskite is 1.34×10^15^ cm^‒3^, which is lower than a 3.43×10^15^ cm^‒3^ of the reference.

**Fig. S28** Dark *I-V* curves of **a** ITO/PEDOT:PSS/BA-based 2D perovskite/spiro-OMeTAD/Au and **b** ITO/PEDOT:PSS/spiro-OMeTAD/Au samples. To study the electrical conductivity variation of VTE-deposited spiro-OMeTAD with and without 2D perovskite, we prepared two samples with structures of ITO/PEDOT:PSS/BA-based 2D perovskite/spiro-OMeTAD/Au and ITO/PEDOT:PSS/spiro-OMeTAD/Au. The dark *I-V* curves are shown as Figure R13. The approximately straight curve indicates the ohmic contact between each layer of the test structure, and the slope represents the total transmission resistance. It is obvious that whether there is 2D perovskite or not, the obtained slope is similar to each other. Therefore, 2D perovskite has no observable negative impact on the conductivity of spiro-OMeTAD.

**Fig. S29** Decay curves of TAS signals at 764 nm for (**a**) neat-PVK, (**b**) PVK/2D and (**c**) PVK/2D/spiro-OMeTAD samples

**Fig. S30** Absorption spectra tracking of perovskite films with and without 2D perovskite

**Fig. S31** Dark *I*–*V* curves of the ITO/PEDOT:PSS/BA-based 2D perovskite/spiro-OMeTAD/Au devices before and after 200 h of aging at 85 °C. To investigate the conductivity stability of the BAI/VTE-deposited spiro-OMeTAD stack under 85 °C, we kept the ITO/PEDOT:PSS/BA-based 2D perovskite/spiro-OMeTAD/Au structure on a hotplate at 85 °C for 200 h. The dark *I*–*V* curves before and after aging are shown in Fig. R14. The nearly linear traces indicate ohmic contacts between all layers, and their slope reflects the total series resistance. No discernible change in slope is observed after aging, demonstrating that the 2D-perovskite/spiro-OMeTAD architecture retains excellent conductivity stability.

**Fig. S32** XRD pattern tracking of (**a**) PVK and (**b**) PVK/2D samples before and after aging

**Table S1** The summary of the PV parameters of perovskite/c-Si TSCs for 0.135 cm^2^ by VTE-deposited and spin-coated spiro-OMeTAD in Fig. 1b

| Parameters | Spin-coated  spiro-OMeTAD | | VTE-deposited  spiro-OMeTAD | |
| --- | --- | --- | --- | --- |
|  | Forward | Reverse | Forward | Reverse |
| *V*_OC_ (V) | 1.892 | 1.914 | 1.912 | 1.927 |
| *J*_SC_ (mA/cm^2^) | 17.75 | 17.82 | 19.02 | 19.09 |
| FF (%) | 78.34 | 80.21 | 79.05 | 80.80 |
| PCE (%) | 26.31 | 27.36 | 28.75 | 29.73 |

**Table S2** The summary of the PV parameters of perovskite/c-Si TSCs (1.01 cm^2^) by VTE-deposited and spin-coated spiro-OMeTAD in Fig. 1b

| Parameters | Spin-coated  spiro-OMeTAD | | VTE-deposited  spiro-OMeTAD | |
| --- | --- | --- | --- | --- |
|  | Forward | Reverse | Forward | Reverse |
| *V*_OC_ (V) | 1.876 | 1.908 | 1.906 | 1.909 |
| *J*_SC_ (mA/cm^2^) | 17.27 | 17.31 | 18.77 | 18.92 |
| FF (%) | 78.19 | 79.10 | 78.94 | 79.67 |
| PCE (%) | 25.33 | 26.13 | 28.24 | 28.77 |

**Table S3** PL lifetimes extracted from the TrPL spectra of the PVK, PVK/2D, PVK/spiro-OMeTAD and PVK/2D/spiro-OMeTAD samples following double exponential fitting

| Samples | *τ*_1_ (ns) | *τ*_2_ (ns) |
| --- | --- | --- |
| neat-PVK | / | 21.14 |
| PVK/2D | 14.68 | 804.9 |
| PVK/spiro-OMeTAD | 15.51 | 186.0 |
| PVK/2D/spiro-OMeTAD | 14.00 | 926.2 |

**Table S4** Time components extracted from the TAS decay curves in Figure S21 for the PVK, PVK/2D, PVK/spiro-OMeTAD and PVK/2D/spiro-OMeTAD samples following double exponential fitting

| Samples | *τ*_1_ (ps) | *τ*_2_ (ps) |
| --- | --- | --- |
| neat-PVK | 5.18 | 282 |
| PVK/2D | 4.35 | 318 |
| PVK/spiro-OMeTAD | 4.81 | 176 |
| PVK/2D/spiro-OMeTAD | 4.29 | 288 |

**Tab. S5** Summarize of device structures and performance parameters in previous studies shown in Fig. 1g

| Structure of the top solar cell in  n–i–p perovskite/c-Si TSCs | Dopant  (HTM) | Method | *V*_OC_ (V) | *J*_SC_ (mA/cm^2^) | FF (%) | PCE (%) | Area (cm^2^) | References |
| --- | --- | --- | --- | --- | --- | --- | --- | --- |
| SnO_2_/MA_0.18_FA_0.74_Cs_0.08_Pb(I_0.94_Br_0.6_)_3_/spiro-OMeTAD/MoO_X_/ITO/ARC | Li-salt and tBP  (spiro-OMeTAD) | Solution processed | 1.78 | 17.8 | 75 | 22.8 | 0.13 | [S7] |
| [SSnO_2_/FA_0.5_MA_0.38_Cs_0.12_PbI_2.04_Br_0.96_/spiro-OMeTAD/MoO_X_/ITO | Li-salt and tBP  (spiro-OMeTAD) | Solution processed | 1.66 | 16.5 | 81 | 22.2 | 0.06 | [S6] |
| SnO_2_/MA_0.18_FA_0.74_Cs_0.08_Pb(I_0.94_Br_0.6_)_3_/spiro-OMeTAD/MoO_X_/ITO/Au  /PDMS | Li-salt and tBP  (spiro-OMeTAD) | Solution processed | 1.75 | 16.9 | 74 | 21.9 | 0.13 | [S8] |
| SnO_2_/MA_0.18_FA_0.74_Cs_0.08_Pb(I_0.94_Br_0.6_)_3_/spiro-OMeTAD/MoO_X_/ITO/Au | Li-salt, Co-salt and tBP  (spiro-OMeTAD) | Solution processed | 1.83 | 16.0 | 70 | 20.4 | 0.13 | [S9] |
| SnO_2_/(FAPbI_3_)_0.83_(MAPbI_3_)_0.17_/spiro-OMeTAD/MoO_3_/ITO/Ag/(Ba,Sr)_2_SiO_4_:Eu^2+^:PDMS | Li-salt, Co-salt and tBP  (spiro-OMeTAD) | Solution processed | 1.73 | 16.5 | 81 | 23.1 | 4.0 | [S10] |
| SnO_2_/Cs_0.17_FA_0.83_Pb(I_0.8_Br_0.2_)_3_/spiro-OMeTAD/MoO_X_/ITO/Au | Li-salt and Tbp  (spiro-OMeTAD) | Solution processed | 1.93 | 16.9 | 74 | 24.2 | 0.86 | [S11] |
| SnO_2_/Cs_0.22_FA_0.78_Pb(I_0.85_Br_0.15_)_3_/spiro-OMeTAD/MoO_3_  /ITO/Au | PTAA, Li-salt and tBP  (spiro-OMeTAD) | Solution processed | 1.85 | 18.0 | 81 | 27.0 | 0.16 | [S12] |
| SnO_2_/(FAPbI_3_)_0.83_(MABr_3_)_0.17_/spiro-OMeTAD/MoO_3_/ITO/Ag | Li-salt, Co-salt and tBP  (spiro-OMeTAD) | Solution processed | 1.82 | 18.1 | 82 | 27.2 | 1.0 | [S13] |
|  |  |  | 1.77 | 18.0 | 76 | 24.2 | 11.8 |  |
|  |  |  | 1.78 | 17.6 | 68 | 21.1 | 65.1 |  |
| SnO_2_/Cs_0.05_MA_0.15_FA_0.8_Pb  (I_0.85_Br_0.15_)_3_/spiro-TTB/TPBI/Vox/IZO/Ag | F6-TCNNQ  (spiro-TTB) | evaporated | 1.83 | 19.5 | 76 | 27.1 | 1.03 | [S14] |
| SnO_2_/ (FAPbI_3_)_0.75_(MAPb  Br_3_)_0.25_ /CPRA/spiro- OMeTAD/MoOx/IZO/Ag | spiro-O  MeTAD(TFSI)_2_  (spiro-OMeTAD) | evaporated | 1.76 | 19.9 | 81.2 | 28.4 | 1.02 | [S15] |
| ALD-TiO_x_/m-TiO_x_/ (FAPbI_3_)_0.75_(MAPb  Br_3_)_0.25_ /poly-TPD/spiro- TTB/MoOx/IZO/Au | Poly-TPD  (spiro-TTB) | evaporated | 1.87 | 19.3 | 83 | 29.9 | 0.16 | [S16] |
| ALD-SnO_X_/KCl/C_60_-SAM/ Cs_0.17_FA_0.83_Pb(Br_0.2_I_0.8_)_3_ /MoO_X_/IZO/Au/MgF_X_ | VNPB  (HTL free) | antisolvent of PVK | 1.89 | 19.8 | 77.9 | 29.2 | 1.04 | [S17] |
| SnO_2_/Cs_0.05_(FA_0.77_MA_0.23_)_0.95_Pb(I_0.77_Br_0.23_)_3_/spiro-OMeTAD/MoOx/IZO/Au | Dopant-free  (spiro-OMeTAD) | evaporated | 1.93 | 19.1 | 80.8 | 29.7 | 0.135 | This work |
|  |  |  | 1.91 | 18.7 | 79.0 | 28.2 | 1.012 |  |

**Supplementary References**

1. P. Caprioglio, M. Stolterfoht, C.M. Wolff, T. Unold, B. Rech et al., On the relation between the open-circuit voltage and quasi-Fermi level splitting in efficient perovskite solar cells. Adv. Energy Mater. **9**(33), 1901631 (2019). <https://doi.org/10.1002/aenm.201901631>
2. R. He, W. Wang, Z. Yi, F. Lang, C. Chen et al. All-perovskite tandem 1 cm^2^ cells with improved interface quality. Nat. Mater. **24**, 252-259 (2023). <https://www.nature.com/articles/s41563-024-02073-x>
3. Q. Zhang, Q. Zhao, H. Wang, Y. Yao, L. Li et al., Tuning isomerism effect in organic bulk additives enables efficient and stable perovskite solar cells. Nano-Micro Lett. **17**(1), 107 (2025). <https://doi.org/10.1007/s40820-024-01613-z>
4. J. Zhang, Y. Hua, B. Xu, L. Yang, P. Liu et al., The role of 3D molecular structural control in new hole transport materials outperforming spiro-OMeTAD in perovskite solar cells. Adv. Energy Mater. **6**(19), 1601062 (2016). <https://doi.org/10.1002/aenm.201601062>
5. G. Du, L. Yang, C. Zhang, X. Zhang, N. Rolston et al., Evaporated undoped spiro-OMeTAD enables stable perovskite solar cells exceeding 20% efficiency. Adv. Energy Mater. **12**(22), 2103966 (2022). <https://doi.org/10.1002/aenm.202103966>
6. Z. Qiu, Z. Xu, N. Li, N. Zhou, Y. Chen et al., Monolithic perovskite/Si tandem solar cells exceeding 22% efficiency *via* optimizing top cell absorber. Nano Energy **53**, 798–807 (2018). <https://doi.org/10.1016/j.nanoen.2018.09.052>
7. S. Zhu, F. Hou, W. Huang, X. Yao, B. Shi et al., Solvent engineering to balance light absorbance and transmittance in perovskite for tandem solar cells. Sol. RRL **2**(11), 1800176 (2018). <https://doi.org/10.1002/solr.201800176>
8. F. Hou, C. Han, O. Isabella, L. Yan, B. Shi et al., Inverted pyramidally-textured PDMS antireflective foils for perovskite/silicon tandem solar cells with flat top cell. Nano Energy **56**, 234–240 (2019). <https://doi.org/10.1016/j.nanoen.2018.11.018>
9. F. Hou, L. Yan, B. Shi, J. Chen, S. Zhu et al., Monolithic perovskite/silicon-heterojunction tandem solar cells with open-circuit voltage of over 1.8 V. ACS Appl. Energy Mater. **2**(1), 243–249 (2019). <https://doi.org/10.1021/acsaem.8b00926>
10. J. Zheng, H. Mehrvarz, C. Liao, J. Bing, X. Cui et al., Large-area 23%-efficient monolithic perovskite/homojunction-silicon tandem solar cell with enhanced UV stability using down-shifting material. ACS Energy Lett. **4**(11), 2623–2631 (2019). <https://doi.org/10.1021/acsenergylett.9b01783>
11. F. Hou, Y. Li, L. Yan, B. Shi, N. Ren et al., Control perovskite crystals vertical growth for obtaining high-performance monolithic perovskite/silicon heterojunction tandem solar cells with VOC of 1.93 V. Sol. RRL **5**(10), 2100357 (2021). <https://doi.org/10.1002/solr.202100357>
12. L. Wang, Q. Song, F. Pei, Y. Chen, J. Dou et al., Strain modulation for light-stable n-i-p perovskite/silicon tandem solar cells. Adv. Mater. **34**(26), e2201315 (2022). <https://doi.org/10.1002/adma.202201315>
13. J. Zheng, W. Duan, Y. Guo, Z.C. Zhao, H. Yi et al., Efficient monolithic perovskite–Si tandem solar cells enabled by an ultra-thin indium tin oxide interlayer. Energy Environ. Sci. **16**(3), 1223–1233 (2023). <https://doi.org/10.1039/D2EE04007G>
14. E. Aydin, J. Liu, E. Ugur, R. Azmi, G.T. Harrison et al., Ligand-bridged charge extraction and enhanced quantum efficiency enable efficient n–i–p perovskite/silicon tandem solar cells. Energy Environ. Sci. **14**(8), 4377–4390 (2021). <https://doi.org/10.1039/d1ee01206a>
15. C. Gao, H. Zhang, S. Ma, H. Su, H. Huang et al., Quasi-conformal monolithic n-i-p perovskite/c-Si tandem solar cells with light management strategies exceed 28 % efficiency. Nano Energy **129**, 110066 (2024). <https://doi.org/10.1016/j.nanoen.2024.110066>
16. L. Duan, S.P. Phang, D. Yan, J. Stuckelberger, D. Walter et al., Over 29%-efficient, stable n–i–p monolithic perovskite/silicon tandem solar cells based on double-sided poly-Si/SiO_2_ passivating contact silicon cells. J. Mater. Chem. A **12**(31), 20006–20016 (2024). <https://doi.org/10.1039/d4ta03396e>
17. M. Zhang, Z. Ying, X. Li, S. Li, L. Chen et al., Hole-selective transparent *in situ* passivation contacts for efficient and stable n–i–p graded perovskite/silicon tandem solar cells. Adv. Mater. **37**(14), 2416530 (2025). <https://doi.org/10.1002/adma.202416530>
